# Nitrogen Uptake in Plants: The Plasma Membrane Root Transport Systems from a Physiological and Proteomic Perspective

**DOI:** 10.3390/plants10040681

**Published:** 2021-04-01

**Authors:** Chiara Muratore, Luca Espen, Bhakti Prinsi

**Affiliations:** Department of Agricultural and Environmental Sciences—Production, Landscape, Agroenergy (DiSAA), Università degli Studi di Milano, I-20133 Milano, Italy; chiara.muratore@unimi.it (C.M.); luca.espen@unimi.it (L.E.)

**Keywords:** plant mineral nutrition, organic nitrogen, metabolic networks, glutamate, subcellular proteomics

## Abstract

Nitrogen nutrition in plants is a key determinant in crop productivity. The availability of nitrogen nutrients in the soil, both inorganic (nitrate and ammonium) and organic (urea and free amino acids), highly differs and influences plant physiology, growth, metabolism, and root morphology. Deciphering this multifaceted scenario is mandatory to improve the agricultural sustainability. In root cells, specific proteins located at the plasma membrane play key roles in the transport and sensing of nitrogen forms. This review outlines the current knowledge regarding the biochemical and physiological aspects behind the uptake of the individual nitrogen forms, their reciprocal interactions, the influences on root system architecture, and the relations with other proteins sustaining fundamental plasma membrane functionalities, such as aquaporins and H^+^-ATPase. This topic is explored starting from the information achieved in the model plant Arabidopsis and moving to crops in agricultural soils. Moreover, the main contributions provided by proteomics are described in order to highlight the goals and pitfalls of this approach and to get new hints for future studies.

## 1. Introduction

Nitrogen (N) is the most abundant mineral element present in plant tissues, in which it constitutes about 1 to 5% of total dry matter [1]. Plants acquire N by roots throughout the life cycle, and the availability of this macronutrient, in terms of total amount and forms, deeply affects plant development and interactions with the environment [2]. In cultivated soils, N availability is a key factor often limiting crop productivity. Hence, there is a worldwide massive use of N fertilizers, despite the detrimental effects on ecosystems and high socioeconomic costs [3]. Improvement of the current knowledge about N nutrition in plants is, therefore, required to reduce the impact of these anthropogenic activities on a global scale.

Leaving aside protein-humic complexes not directly bioavailable to plants, in soil N is present as inorganic forms, such as nitrate (NO_3_^−^) and ammonium (NH_4_^+^), and as organic forms, mainly consisting of urea, free amino acids, and short peptides. The accessibility of these resources by roots varies considerably through space and time, due to soil heterogeneity and to dynamic microbial conversions, two aspects in turn affected by agronomic practices and environmental conditions [4]. In aerobic soils, NO_3_^−^ is the most abundant form, with concentrations between 1 to 5 mM, while NH_4_^+^ concentration typically ranges between 20 and 200 μM. However, NO_3_^−^ is readily leached, while NH_4_^+^ is strongly adsorbed by soil particles and slowly released [5]. Differently, free amino acids and urea are generally present in concentrations ranging from 1 to 150 µM and <70 µM, respectively [5,6,7], representing a minor proportion of the available N for crops.

The relevance of organic N for crop nutrition is a matter of debate. Although there is evidence that plants can acquire amino acids, small peptides, and (partial) proteins from the soil [8], a high impact in agricultural contexts was traditionally ruled out [5,9]. However, in recent years, many studies opened new questions. First observations proved that the supply of sole amino acids sustains plant growth, and were soon followed by the characterization of root transport systems for amino acid uptake, whose molecular bases were partly elucidated in *Arabidopsis* (*Arabidopsis thaliana* L.) and confirmed in crops [10,11]. At the same time, it was proven that the provision of amino acids, even at a very low concentration, can affect root morphology and plant growth [12]. Similarly, considering that urea-based formulations account for over 50% of total N fertilizers applied in agriculture, the discovery of urea transporters in the plasma membrane (PM) of root cells has drawn attention to a direct use of this nutrient by plants [13].

Total N availability, and the forms supplied, affect seed germination, plant growth, root and leaf functionalities, hormonal balance, and seed production. Recent literature stresses the importance of interpreting N nutrition as a composite scenario, taking into account the contribution of individual nutrients, their reciprocal interactions, and the plethora of effects on plant metabolism [14,15]. Needless to say, the “-omic” approaches, given their intrinsic holistic nature, seem to adequately respond to this need.

**Figure 1:**
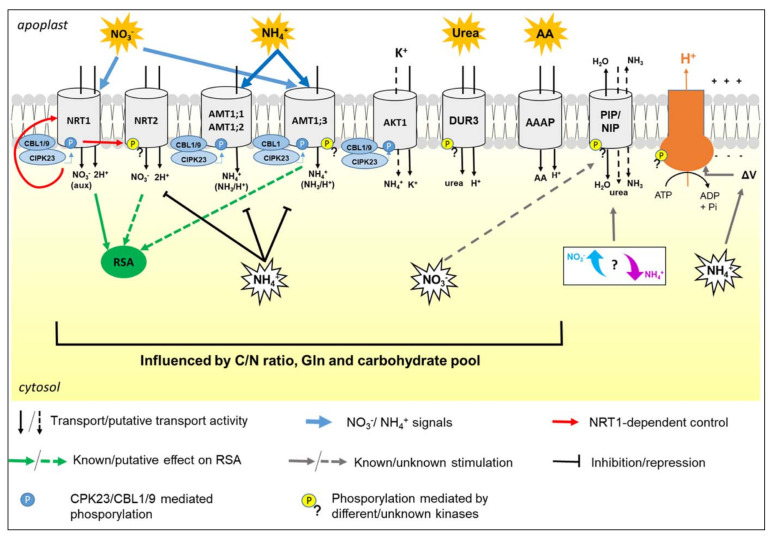
The figure summarizes the regulatory pathways and the metabolic relations among the main transporters involved in N uptake by roots, taking as a model Arabidopsis. For simplicity, transporters, transceptors, aquaporins, and H^+^-ATPase are reported in the same root cell, but it is not the common real case. Acronyms and details are explained in the text.

In roots, the major adaptations to N availability consist of the changes in uptake activity and in the modulation of the root system architecture (RSA), both of which are related to the ability of N forms to act as nutrients and/or regulatory signals for plant growth and metabolism (Figure 1).

Transport and sensing of N forms involve proteins located in the PM of root cells. In recent years, there was a huge increase in knowledge about the control of uptake at the transcriptional level, as well as about the components involved in signaling. It was also highlighted that post-translational modifications (PTMs) of transporters, such as phosphorylation events and formation of protein complexes, have key roles in rapid adaptations to sudden changes in N availability [11,13,16,17].

From a physiological perspective, N uptake is also related to other main PM activities, such as the formation of the electrochemical proton gradient and water homeostasis. Hence, this review provides an overview of N nutrition in plants, trying to integrate the notions about single N forms, their interactions, as well as the relations with other PM functionalities (Figure 1).

Many studies have been focused on the identification of proteins involved in N uptake, reaching the most complete molecular characterization in Arabidopsis. We used Arabidopsis as a starting reference point, but several parallelisms in crops are reported. However, the description of these aspects in legumes (*Fabaceae* spp.) and actinorhizal crops, as well as in rice (*Oryza sativa* L.), is out of our scope due to their numerous peculiarities.

This topic is examined describing the contribution of proteomics, in order to highlight goals and pitfalls and to get new hints for future studies. Deciphering how the root membrane proteome changes in response to different N sources could provide new knowledge useful to enhance agriculture sustainability.

## 2. Transporters and Transceptors Involved in Nitrogen Uptake by Roots

The uptake of N nutrients is finely controlled and influenced by the interplay among three main protein classes, namely transporters, receptors, and transceptors. In this review, the term transporter is used as a synonym of selective carrier proteins, while the term transceptor refers to proteins able to fulfil a dual transport/sensing function [18]. Finally, it is worth remembering that the total concentrations of N forms in root cells depend on external availability as well as on the balance among net uptake (defined as the difference between influx and efflux), root metabolization, vacuolar accumulation, and xylem/phloem (un)loading [1].

### 2.1. Nitrate Uptake

Nitrate is the primary N source for plant growth in most agricultural soils, and its availability significantly affects crop productivity [9]. At high external supplies, total NO_3_^−^ concentration in root cells can reach up to 100 mM, mostly stored in the vacuole, while cytosolic concentration is kept low, as measured by selective-microelectrodes in cereals [19]. Electrophysiological studies indicated that NO_3_^−^ uptake by roots is always an active process, mediated by a 2H^+^/1NO_3_^−^ symport mechanism, while NO_3_^−^ efflux is passive, saturable, and inducible [20,21].

In plants, NO_3_^−^ uptake is mediated by transporters of the NPF family (previously named NRT1/PTR family) and of the NRT2 family, which in Arabidopsis consist of 53 and 7 members, respectively [22,23]. Although there are no sequence homologies between the two families, these transporters share the same topology consisting of 12 transmembrane domains and have both the N- and C- termini lying on the cytosolic side of the membrane [24]. The activity of these proteins is strictly regulated by NO_3_^−^ availability and plant N nutritional status. In N starved plants, renewed availability of NO_3_^−^ triggers the typical NO_3_^−^ primary response (NPR), which comprises the rapid induction of NO_3_^−^ transporters and N assimilating enzymes. This adaptation is followed by later down-regulation of uptake, correlated with the accumulation of NO_3_^−^ itself and of its downstream metabolites, such as glutamine (Gln, Figure 1) [25]. A prominent role for the transcriptional control of NO_3_^−^ transporters was demonstrated in Arabidopsis and crops, and the importance of PTMs was also highlighted [26,27,28].

In Arabidopsis, three members of the NPF family are located in the PM of root cells and participate in NO_3_^−^ uptake (Table 1).

NRT1.1 (AtNPF6.3) and NRT1.2 (AtNPF4.6) participate in NO_3_^−^ influx, and the second is the major one responsible for the constitutive influx in the low-affinity range (>0.25 mM) [33], while NAXT1 (AtNPF2.7) mediates NO_3_^−^ efflux to the external medium [34].

**Figure 2:**
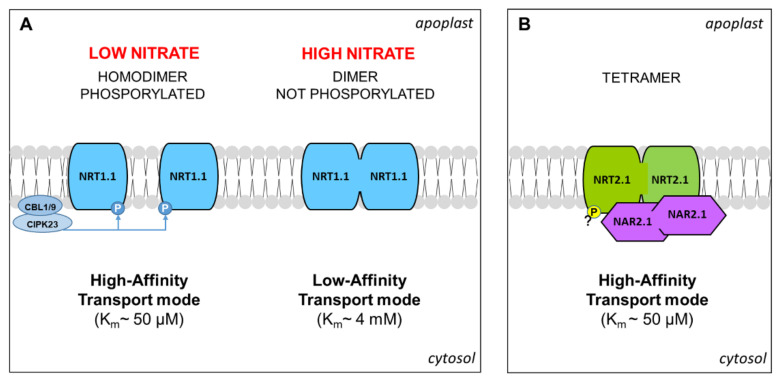
The model proposed for the NRT1.1 dimerization mediated by phosphorylation (adapted from [47]) (**A**). Model proposed for the tetramer NRT2.1/NAR2.1 according to [22,50] (**B**). Details are explained in the text.

The characterization of NRT1.1, as the first transceptor identified in higher plants, represents a milestone in plant nutrition research [29]. NRT1.1 is an inducible dual-affinity transporter able to participate in the uptake of NO_3_^−^ both in the low-affinity (Km~4 mM) and high-affinity (Km~50 µM) ranges, depending on the dephosphorylation/phosphorylation of threonine (Thr)-101 [30]. Interestingly, the determination of the crystal structure of NRT1.1 suggested that phosphorylation also influences the oligomerization state of the protein in the PM. According to the proposed model [47], at high external concentrations of NO_3_^−^, the absence of phosphorylation of Thr-101 permits that NRT1.1 forms dimeric complexes operating in low-affinity mode. Instead, when NO_3_^−^ concentration is low (<0.2 mM) [30], the phosphorylation of NRT1.1 determines dimer decoupling, by which individual protomers adopt a high-affinity transport mode. The phosphorylation of Thr-101 is mediated by CIPK23 kinase (CBL-interacting serine/threonine-protein kinase 23). In detail, CIPK23 is recruited at the PM by binding with one of the two proteins CBL9 or CBL1 (calcineurin B-like 1 or 9), which also enables its activation by auto-phosphorylation (Figure 2A) [48].

According to its definition as a transceptor, NRT1.1 plays a key role as an NO_3_^−^ sensor, showing a signaling function that is independent of the transport activity. NRT1.1 regulates a very ample set of plant responses to NO_3_^−^, by a signaling cascade which is partly elucidated [16]. At the root level, these responses include both changes in RSA (Section 3) and the regulation of NO_3_^−^ uptake (Figure 1). Once again, the functionality of NRT1.1 as an NO_3_^−^ sensor (and of its concentration) depends on its phosphorylation state. For instance, the phosphorylated and not-phosphorylated forms participated in the short-term biphasic up-regulation of the *NRT2.1* gene (see below) in response to low and high external NO_3_^−^ inputs, respectively, while the phosphorylated one mediates the long-term down-regulation of NRT2.1 at high NO_3_^−^ concentrations [29,49].

Among the NRT2 family in Arabidopsis, four members were characterized as responsible for the high-affinity influx of NO_3_^−^ in roots (<0.25 mM, Table 1). NRT2.1 and NRT2.2 are the major components of the inducible HATS (High Affinity Transport System), being highly induced at the transcriptional level in roots by exposure to NO_3_^−^ [36]. NRT2.4 and NRT2.5 are characterized by a very high-affinity and are strongly induced by N deprivation but rapidly repressed by NO_3_^−^ and NH_4_^+^, suggesting their primary role in the earliest influx of NO_3_^−^ after starvation [37,38,51].

The molecular mechanisms governing NRT2.1 were largely described. The transcription of the *NRT2.1* gene is rapidly induced by NO_3_^−^ in an NRT1.1-dependent manner (see above), at a low or high level according to the concentration of the anion [29]. Moreover, it is repressed by Gln and NH_4_^+^, and diurnal regulated, probably by a carbohydrate pool translocated from the shoot (Figure 1) [24]. In addition, NRT2.1 was proposed as a potential NO_3_^−^ transceptor, involved in the modulation of RSA (Section 3). The recruitment of NRT2.1 to the PM requires binding with the protein NAR2.1 (Nitrate Assimilation Related protein), forming a stable tetramer composed of two subunits for each protein (Figure 2B) [50]. However, in Arabidopsis, the dependency of functionality on NAR2.1 was demonstrated for NRT2.5 and excluded for NRT2.4 [37,51], but the physiological meaning is not yet elucidated. Interestingly, in recent years, some proteomic investigations contributed to discovering additional molecular events that regulate NRT2.1. First indications were provided by a phosphoproteomic study aimed at the characterization of the early changes induced by NO_3_^−^ or NH_4_^+^ resupply to Arabidopsis seedlings, based on the phosphopeptide enrichment by the titanium dioxide methodology. This approach revealed that NRT2.1 is phosphorylated in serine (Ser)-28 in N starved seedlings and is rapidly dephosphorylated after resupply of high NO_3_^−^ concentrations, suggesting that this mechanism contributes to inactivate NRT2.1 when the activity of low-affinity transporters becomes predominant [52]. Later on, it was clarified that the phosphorylation at Ser-28 contributes to increase the stability of NRT2.1 and is required for the accumulation of this protein in response to low NO_3_^−^ availability [53]. Furthermore, by a phosphoproteomic approach specifically devoted to NRT2.1 characterization, it was recently proven that NRT2.1 can be also phosphorylated in the C-terminus (at Ser-510). This PTM leads to NRT2.1 inactivation in response to high N supplies, without affecting its interaction with NAR2.1, proving a novel and fundamental mechanism for the regulation of NO_3_^−^ uptake in Arabidopsis roots [54].

Although the huge amount of information obtained in Arabidopsis is exciting, it is important to remember that direct parallelisms in crops must be done with caution. For instance, a recent characterization of ZmNPF6.6, an NRT1.1 homolog in maize (*Zea mays* L.), confirmed the induction by NO_3_^−^ and the involvement in its low and high-affinity uptake. However, the biphasic kinetic, the regulation by phosphorylation, and the auxin transport were not confirmed [55]. Similarly, the literature indicates that in crops the binding of NRT2 could involve different members of the NAR family, since partner pairs are species-dependent [56].

### 2.2. Ammonium Uptake

Ammonium is an important N nutrient rapidly absorbed and assimilated by plants, as well as a signaling molecule influencing plant growth and RSA (Section 3). However, many crops are sensitive to NH_4_^+^ toxicity, especially at high dosages, and generally results in stunted growth, leaf chlorosis, and poor root development. Indeed, for optimal growth most crops require the contemporaneous availability of NO_3_^−^ and NH_4_^+^, even if the best ratio depends on plant species, developmental phase, and environmental conditions [9,57].

In plants, the uptake of NH_4_^+^ and its allocation among organs are highly influenced by nutritional conditions. At the cellular level, it was estimated that NH_4_^+^ concentrations in cytosol and vacuole range from 1 to 10 mM and 1 to 45 mM, respectively [58], while, interestingly, in roots, the apoplastic NH_4_^+^ concentration is buffered around 1 to 2 mM, both under low and high NH_4_^+^ supplies [40].

At higher external NH_4_^+^ concentrations, there is evidence that the nutrient probably permeates into root cells both as NH_3_ and NH_4_^+^ through two distinct mechanisms. The first one is the passive and electroneutral influx/efflux cycle of NH_3_, putatively facilitated by aquaporins (Section 4.1), that results in the hyper-accumulation of the charged form into the vacuole. The second one includes the NH_4_^+^ influx likely mediated by other PM channels, such as non-selective cation (NSCC) and potassium (K^+^) specific channels, among which AKT1 (Figure 1) [57,59].

At low external NH_4_^+^ concentrations (<1 mM), NH_4_^+^ influx is a saturable and highly controlled process, mediated by members of the AMT1 subfamily (of the Ammonium Transporter/Methylammonium Permease family) through NH_4_^+^-uniport or NH_3_/H^+^ co-transport. These are 45 to 65 kDa proteins with 11 hydrophobic transmembrane domains, a cytosolic C-terminus and an N-terminus in the apoplast [58,60,61]. In Arabidopsis, four members of the AMT1 family are responsible for high-affinity NH_4_^+^ (<1 mM) uptake in the roots (Table 1).

In detail, AMT1;1, AMT1;3 and AMT1;5 are accumulated in the epidermis and root hairs, which contribute in an additive manner to NH_4_^+^ uptake via the symplastic route [39,40]. However, the different substrate affinities suggest that AMT1;1 and AMT1;3 (K_m_ ~50 and 60 µM, respectively) mainly operate at NH_4_^+^ concentrations common in soil, while AMT1;5 (K_m_ ~5 µM), which is accumulated only under prolonged N starvation, might significantly contribute when the availability of NH_4_^+^ is very low [40]. Finally, since AMT1;2 has a lower affinity (K_m_ ~230 µM) and is accumulated in the endoderm, it is thought to be mainly involved in the retrieval of NH_4_^+^ that enters root through the apoplastic route [40].

At the transcriptional level, the *AMT1* genes are generally subjected to diurnal changes, probably regulated according to the rate of carbohydrate translocation from the shoot. Moreover, *AMT1* gene expression is induced during N starvation and reduced under high NH_4_^+^ availability, probably through mechanisms exerted by its downstream metabolites, such as Gln (Figure 1) [40,58,62].

**Figure 3:**
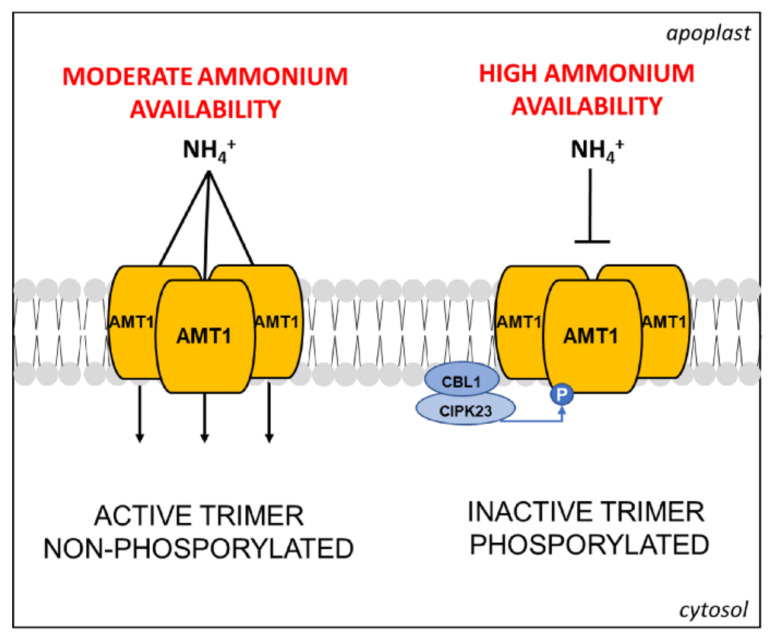
Schematic representation of the AMT1 trimeric complex and its control mode through CIPK23/CBL1-mediated phosphorylation induced by high NH_4_^+^ availability, adapted from [69] (see text for further details).

At the protein level, these transporters form trimeric complexes localized in the PM [63], for which post-translational regulations were suggested already from the earliest molecular studies [62]. In recent years, several proteomic investigations have significantly contributed to elucidate this aspect, pointing out the key role of phosphorylation at the C-terminus in AMT1 subunits. Firstly, a phosphoproteomic study, conducted by IMAC methodology (Ion Metal Affinity Chromatography) on PMs enriched fraction from cell suspensions of Arabidopsis, identified a phosphorylation site in the C-terminus of AMT1 conserved in three members of the family [64]. Then, studies on AMT1;1 and AMT1;2 proved that the C-terminus of the AMT1 subunit acts as an allosteric regulator of the complex. According to the proposed molecular model, in the non-phosphorylated form, the C-terminus interacts within its own monomer and with the adjacent one, assuring the transport. Its phosphorylation (at Thr-460 in AMT1;1) leads to *trans*-inactivation of the whole complex (Figure 3) [63,65].

Later on, a dedicated study on Arabidopsis roots was conducted combining phosphoproteomics with complementary analyses, such NH_4_^+^ pulse treatments, determination of ^15^N-NH_4_^+^ uptake, and protein blots [66]. This combined approach proved that phosphorylation of Thr-460 in AMT1;1 is induced by NH_4_^+^ in a time- and concentration-dependent manner, while neither NO_3_^−^ nor Gln (nor endogenous nor external) trigger this response, overall proving a feedback mechanism able to tune NH_4_^+^ uptake capacity to prevent toxicity [66]. The same study proposed that AMT1;1 could act as a transceptor, but this hypothesis was successively questioned. For instance, a comparative study of proteomic and transcriptomic profiles of Arabidopsis roots within three hours of NO_3_^−^ or NH_4_^+^ deprivation indicated that the dephosphorylation of AMT1;1 was not concomitant with large transcriptomic changes, suggesting that the predominant regulative signal is the endogenous NH_4_^+^ concentration [67]. However, the same study paved the way to discover the putative roles of AMT1;3 as the transceptor involved in the modulation of RSA (Section 3).

Other studies revealed an additional degree of complexity in the NH_4_^+^ uptake regulation. In Arabidopsis roots, it was shown that AMT1;1 and/or AMT1;3 interact in functional homo- and heterotrimers, both subjected to (*trans*)-inactivation exerted by the phosphorylation of the AMT1;3 subunit [68]. The same study has also put in evidence that the phosphorylation in AMT1;3 does not affect the functionality of AMT1;2 or AMT1;5, suggesting different regulative pathways for individual AMT1 transporters. In the same years, the above-cited proteomic investigations on NO_3_^−^/NH_4_^+^ resupply or deprivation in Arabidopsis identified novel phosphorylated sites in the C-terminus of AMT1;1 and AMT1;3 differently modulated in response to NO_3_^−^ [52,67]. The molecular network behind was partly elucidated a few years ago. Firstly, a large-scale genetic screening in Arabidopsis led to the discovery that the phosphorylation at the C-terminus of AMT1;1 and AMT1;2 is catalyzed by the CIPK23/CBL1 complex in response to high NH_4_^+^ availability, although the involvement of other kinases was not excluded (Figure 3) [69].

In Arabidopsis the CIPK23/CBL1/9 complex is also involved in the positive regulation of the K^+^ transporter AKT1 [48], which in barley (*Hordeum vulgare* L.) was suggested as one of the unspecific low-affinity importers of excess NH_4_^+^ [70]. From a physiological point of view, this mechanism is very interesting because it could contribute to enhancing the NH_4_^+^ uptake, and hence N resources for plant growth, when the risk of toxicity is avoided by the co-presence with NO_3_^−^. Moreover, this novel scenario highlights the interplay of the molecular pathways controlling the uptake of NO_3_^−^, NH_4_^+^, and K^+^ (Figure 1). Proteomic studies highlighted a major accumulation of the K^+^-transporter HAK5 in roots of Arabidopsis plants when grown in NH_4_^+^ instead of NO_3_^−^, suggesting a compensatory response [67]. Similarly, in maize and potato roots, an increase in the accumulation of the voltage-gated potassium channel beta subunit was observed in response to NO_3_^−^ availability [71,72].

The study of Straub and co-workers also revealed that the phosphorylation of AMT1;3 (Thr-464) is mediated by a kinase not yet identified, evidencing the involvement of different kinases in the regulative pathway of AMT1 [69]. Moreover, it was recently demonstrated that AMT1;3 can be phosphorylated in additional sites (Ser-480, Ser-487, Thr-494) in response to NO_3_^−^ availability (Figure 1). In particular, the activity of AMT1;3 (dephosphorylated in Thr-464) can be further increased by dephosphorylation of Thr-494 [73]. Overall, these observations point out how much the NH_4_^+^ uptake is finely regulated, proving its relevance in plant physiology.

The molecular determinants of NH_4_^+^ uptake are generally conserved across an ample set of plant species, but significant differences were reported in the transcriptional regulation of AMT1 among crops [17]. For instance, in several cereals, the supply of NH_4_^+^ triggers an increase in the NH_4_^+^ influx, which is kept stable for a few hours (even for 24 h in maize) before the feedback negative regulation occurs. In maize, this response was associated with a peculiar induction of ZmAMT1;1 and ZmAMT1;3 triggered by a local NH_4_^+^ signal, independent from plant nutritional status, but the complete elucidation of the regulative events is not yet elucidated [74]. Overall, considering the ample range of degrees in adaptability/sensitivity to NH_4_^+^ of crops and cultivars [9], the study of NH_4_^+^ nutrition in plants is still an open, and partially unexplored, research field.

### 2.3. Uptake of Amino Acids

In plant tissues, the content of total amino acids usually ranges between 1 and 10 mM, but this value is very variable and can highly increase, especially under high NH_4_^+^ nutrition [9]. Similarly, the distribution of total amino acids in plant cell organelles is variable, with the highest concentration typically found in plastids and cytosol [75,76]. In plant tissues, it is possible to distinguish the group of the “major” amino acids (i.e., more abundant, including glutamate (Glu), Gln, aspartate, asparagine, alanine (Ala)) and the group of the “minor” ones, present at lower levels and whose biosynthesis is subjected to a strong end-product feedback control [77].

As previously stated, amino acid concentration in soils ranges between 1 and 150 µM and, once again, the “major” amino acids are the most abundant [7]. The soil amino acid content is highly influenced by environmental conditions, microorganism activities, and soil buffer capacity that renders basic amino acids (arginine (Arg), lysine (Lys), and histidine (His)) less mobile and less available for roots [5,9]. However, considering the intense exchanges of organic exudates between microorganisms and roots, it is possible that the amino acid compositions in the rhizosphere and in bulk soil significantly differ [10].

The effectiveness of amino acids as N nutrients for plants is a controversial issue. The major objections reside in the low diffusion coefficient of amino acids in the soil as well as in their short half-life (about 4 h), two factors that could limit the competitiveness of plants versus microorganisms in the uptake of these compounds [9]. On the other hand, there is increasing evidence that plants acquire amino acids from the growing media, also when both NO_3_^−^ and NH_4_^+^ are available [7]. Physiological studies on crops provided contradictory results. For instance, barley plants, grown in hydroponics and supplied with amino acid concentrations similar to field conditions, showed an uptake rate with Michaelis-Menten kinetics [78]. Conversely, a ^14^C/^15^N tracer study in maize seedlings, grown in rhizosphere tubes filled with soil and treated with Ala or NO_3_^−^, showed a scarce relevance of organic N uptake [79]. In our opinion, some aspects should deserve specific attention. Firstly, the studies conducted in hydroponics or in experimental media generally provide results supporting the nutritional value of amino acids, while those conducted in soil conditions give opposite results. It is, therefore, plausible that some results are biased due to the interferences of soil particles and to the experimental set-up that somehow could modify the expected amino acid bioavailability. Secondly, the provision of a single amino acid could be misleading because one amino acid is not the other, nor mimics the soil chemical composition.

In Arabidopsis, amino acids prompt different effects when provided at relatively high concentrations (3 mM). Some of the “major” amino acids promote plant growth, Glu has no effects, while the supply of Arg or Ala is positive singly but negative when in combination with NO_3_^−^. Finally, the provision of valine (Val), Ser, and isoleucine (Ile) seem to be detrimental to plant growth [80]. These observations support the use of amino acid by plants, hint interlinks with the inorganic N nutrients, and suggest that “minor” amino acids, when provided in high concentrations (not common in soil), could be toxic due to metabolic interferences [10].

Some high-affinity amino acid transporters, with kinetics comparable to those of microorganisms, have been characterized in the last years [10,81]. In the Arabidopsis genome, more than 100 genes encoding for amino acid carriers were predicted, which belong to the amino acid/polyamine/organocation (APC) superfamily [82] and to the UMAMIT (Usually Multiple Amino acids Move In And Out Transporters) group of the drug/metabolite transporter superfamily [83]. Considering the complexity of amino acid metabolism in plants, the tissue specificity and cell localization of these transporters is fundamental to define their actual physiological functions [11,77].

To date, five carriers that were characterized as amino acid importers in roots of Arabidopsis, all comprised in the AAAP (amino acid/auxin permease) family of the APC superfamily (Table 1). Gene expression in heterologous systems together with complementation studies in yeast characterized these carriers as electrogenic H^+^/amino acid symporters. Moreover, the structural analyses of AAP1 (Amino Acid Permease 1), taken as a model, showed that these proteins contain 11 trans-membrane domains with the N-terminus inside the cytoplasm and the C-terminus facing the outer PM surface [84]. The characterization of Arabidopsis mutants fed with amino acids at low concentrations, together with β-glucuronidase and GFP-tagging studies, provided information about the functionalities of these carriers in plants, their expression patterns, and their location in the PM. In detail, LHT1 (Lysine Histidine Transporter 1) is a high-affinity carrier involved in the uptake of neutral amino acids, His, and acidic amino acids, located in the root and leaf cells [41,42]. AAP5 mediates 68 to 88% of the uptake of Lys and Arg in the high-affinity range and, despite being present in all plant tissues, is preferentially accumulated in the root cortex [42,43]. In addition, LHT6, a carrier localized in root hairs, epidermis, cortex, and endodermis, participates in the high-affinity uptake of acidic amino acids, Ala, Gln, and probably phenylalanine (Phe). The same authors also proposed that AAP1, localized in cotyledons and roots, could mediate the high-affinity uptake of Ala, Gln, proline (Pro), Ser, and Glu, although the need for verification was stated [7,44]. Finally, ProT2 is an importer of Pro and glycine betaine located in the root epidermis and cortex, but in Arabidopsis the activity in the high-affinity range (500 µM) was proven only for the second compound [45].

Amino acid efflux is fundamental in the rhizosphere relations, as demonstrated by its change in response to microbial compounds [85] and nutrition limitations [86]. However, its quantitative significance in normal conditions was doubted by several authors, which ascribed it to a small leakage down a concentration gradient [10]. The analyses of root exudate in several crops in non-axenic condition indicated a predominant extrusion of Gly, Ser, and Ala, supporting the involvement of selective transport systems [87]. In recent years, the UMAMIT family was characterized as a class of facilitators with bidirectional properties, and several members are expressed in roots [11], but to date members specifically involved in amino acid exports into the soil are not yet identified [88].

Considering this framework, it is highly conceivable that these transporters have complementary roles in amino acid uptake from the soil, with individual contributions probably varying during plant development and growth conditions. Additionally, the presence of LHT1 and AAP5 in the aerial organs [41,43], as well as the localization of AAP1 in the cotyledon vasculature [44], supports their involvements in other processes of amino acid transport and allocation throughout the plant. For instance, in seedlings *LHT1* is expressed in the root epidermis, but it is confined to the root tips in older plants (Table 1). Similarly, *AAP5* is highly expressed in roots of young plants [89], but in older plants, its expression seems to become predominant in aerial organs [90]. From these considerations, it was proposed that the contribution of LHT1 and AAP5 in amino acid uptake by roots is substantial during the early developmental stages but could become indirect later [91]. Currently, there is no evidence for the existence of amino acid transceptors in plants [92].

In Arabidopsis seedlings, the expression of *LHT1* was found to be inducible by amino acids (5 mM) and by NO_3_^−^ (5 mM) if compared to plants supplied with NH_4_NO_3_ (40 mM) [41]. Although these results are indicative, the high concentrations used do not allow to predict the responses in field conditions. Differently, *AAP1* resulted to be induced by light, sugars, amino acids, NO_3_^−^, and also by NH_4_^+^. The studies on ProT2 were mainly focused on its induction by water and salt stress, which was confirmed in different crops, but little is known about the effects of N nutrients [93]. Gene expression analysis of *LHT1*, *AAP1,* and *ProT2* in Arabidopsis root and shoot in response to NO_3_^−^ induction revealed different regulation depending on timings, doses, and organs. In the long-term (24 h), *AAP1* resulted lowly induced equally in both organs, *ProT2* seemed slightly de-induced and *LHT1* was highly induced, especially in shoots [93]. This scenario depicts the complexity of studying these genes in the context of plant N nutrition (Figure 1). As highlighted by the literature [7], a major limitation consists in the different roles in roots (i.e., amino acid uptake from the soil) and shoots (i.e., supply of amino acids to mesophyll cells from the xylem sap). These functionalities could differently respond to N inputs as well as could be differently affected by factors, including photosynthetic activity and energy metabolism, with a final outcome that is very challenging to discern the regulatory network. However, an increasing interest is directed to the AAAP family, and, in our opinion, recent genome-wide analyses, such as those conducted in maize [94], potato (*Solanum tuberosum* L.) [95], tobacco (*Nicotiana* spp) [96], and wheat (*Triticum aestivum* L.) [97] are paving the way to unravel this issue in the coming years. On the contrary, proteomics was very rarely applied in the field of amino acid nutrition in plants. To our knowledge, two research projects were conducted about the responses to Gly-based nutrition in *Lolium perenne* and *Brassica campestris* [98,99]. Although these pioneering studies revealed new information about Gly metabolism in plants, no amino acid transporters were detected among the responsive proteins. This lack is in part attributable to the 2-DE based analytical approach adopted, known to be unsuitable for resolving membrane proteins (Section 5, Table 2). Indeed, very recently an iTRAQ-based study (Isobaric Tags for Relative and Absolute Quantitation) allowed to characterize LHT1, confirming its key role in N remobilization in Arabidopsis seedlings [100]. That hints that the right time has come for proteomics to help unravel the complex metabolic network behind amino acid nutrition in plants. Future studies could obtain new information useful to ameliorate soil management and agricultural sustainability.

### 2.4. Urea Uptake

In the last decades, the use of urea fertilizers is increased to account for more than 50% of the world N fertilizer applications, mainly thanks to low costs and high N content. In soil, urea is rapidly hydrolyzed by ubiquitous microbial urease with the release of NH_3_, subsequently converted into NO_3_^−^ by nitrifying bacteria [101]. Therefore, in agricultural soil, urea concentration is generally low (up to 70 µM) and it was thought for a long time that this kind of fertilizer provided N to crops mainly in the form of NH_4_^+^. However, the identification of a dedicated high-affinity transporter led to the re-evaluation of urea as a direct N source [102]. In addition, urea is a key molecule in N translocation and recycling in plants. In source tissues, urea is produced by Arg catabolism and is hydrolyzed by cytosolic urease to release NH_4_^+^, which is then re-assimilated in sinks. In this view, the presence of endogenous urease activity in almost all plant tissues reinforces the hypothesis that plants can use the molecule as a nutrient [103].

The first urea transporter identified in higher plants was DUR3 in Arabidopsis, a high-affinity urea/H^+^ symporter that belongs to the SSS (Sodium-Solute Symporters) family (Table 1). AtDUR3 is an integral protein with 14 transmembrane domains and the N- and C- termini protruding into the outer side of the membrane [104]. In Arabidopsis roots, DUR3 localizes to the PM and sustains up to 90% of urea influx in the high-affinity range (K_m_ ~4 µM), while the urea influx in the low-affinity range was ascribed to diffusion throughout aquaporins (Section 4.1) [46].

When provided as the sole N source in axenic conditions, urea is taken up as an intact molecule, partly translocated to the shoots, and partly assimilated into amino acids, despite being less efficient than inorganic N forms. These metabolic responses are associated with an increase in expression of At*DUR3* and of genes involved in amino acid metabolism and transport [105]. Interestingly, the urea contents in roots scarcely differ in plants supplied with urea or with inorganic N, suggesting that the endogenous urease activity is sufficient to metabolize the urea taken up from growing media [46,103,105].

The expression of the At*DUR3* gene is high during N starvation, dramatically induced further after resupply of urea, but repressed after resupply of NH_4_^+^ or NO_3_^−^. GUS-promoter analyses showed that the expression of At*DUR3* significantly increases during N starvation in the epidermis and cortex, and to a lesser extent in the vasculature tissues near the xylem (Table 1; Figure 1) [46].

Physiological and transcriptomic studies in Arabidopsis plants confirmed that urea uptake is stimulated by substrate and reduced when inorganic N is available [105]. Further studies in maize and wheat agree in the observation that co-provision of urea and NO_3_^−^ has overall positive effects on plant growth and N use. The explanation resides in a reprogramming of the assimilation pathways that seems to assure a better metabolic balance [106]. This behavior is very similar to what is described under the co-provision of NO_3_^−^ and NH_4_^+^ by physiological [9] and proteomic studies. For instance, a recent characterization of proteomic profiles in roots and leaves of maize showed that the co-provision of NO_3_^−^ and NH_4_^+^ is related to specific changes in the abundance of enzymes involved in C and N metabolism, water balance, and stress responses [71]. In the future years, it will be of interest to investigate distinct signatures between urea and NH_4_^+^ in co-presence with NO_3_^−^.

To date, DUR3 orthologues were identified in several crops, indicating that higher plants have only a single *DUR3* gene [13]. The functional characterization of DUR3 in maize confirmed the main features of its involvement in urea uptake in roots [107] and suggested a role in urea vascular loading in leaves [108]. Interestingly, a recent study in tomato revealed different *DUR3* transcript abundances in cultivars showing different nitrogen use efficiency (NUE) [109].

To the best of our knowledge, no proteomic study investigated urea nutrition in plants. However, Menz and co-workers detected that in Arabidopsis, DUR3 was more abundant under NH_4_^+^-adapted conditions [67]. Although this result is apparently in contradiction with *DUR3* expression studies, it could suggest possible changes in Arg catabolism after long exposure to NH_4_^+^. Even more interestingly, these authors detected phosphorylation of Ser-568 in DUR3 which showed a threefold increase after 15 min of NH_4_^+^ depletion and a decrease after 3 h. So far, the influence of this PTM on DUR3 functionality was not yet further investigated.

Overall, new perspectives about the roles of urea in plant nutrition have been proposed in the last years. However, a better understanding of the physiological and biochemical interplay among urea, NO_3_^−^ and NH_4_^+^ is still required. In addition, some environmental concerns exist about possible urea runoff from the soil following the massive use of urea fertilizers, especially if in combination with urease inhibitors [101]. This aspect could be further exacerbated considering that urease inhibitors can negatively affect urea metabolism in plants [110]. Therefore, future studies are needed to reach a fully aware use of urea in agriculture.

## 3. Influences of Nitrogen Forms on Root System Architecture

**Figure 4:**
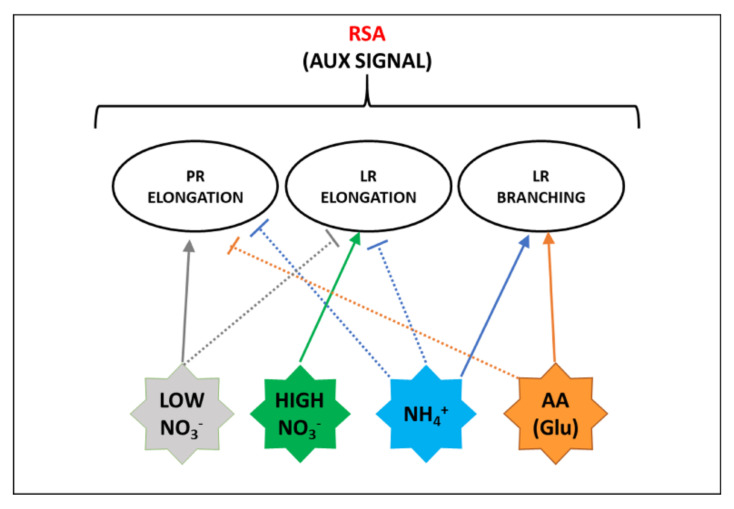
Scheme of the main effects of the different N forms on the responses affecting root system architecture (RSA) that are also related to auxin (aux) signal. Full lines indicate positive effects, dashed lines indicate negative effects. Details are explained in the text.

The root system architecture (RSA) is defined as the three-dimensional spatial configuration of root components within the soil. The primary root (PR) length defines the depth of the vertical soil layers reached by the plant, while lateral roots (LR) enhance horizontal soil exploration. In addition, root hairs greatly expand the absorptive surface area [111,112]. The arrangement of these components of the root system is determined by the interplay between developmental genetic programs and (a) biotic environmental cues [112,113]. RSA shows high plasticity according to both plant endogenous nutritional status and to the kind of N form available in growing media (Figure 4).

Root architectural responses to NO_3_^−^ supply are strictly dependent on the availability and spatial/temporal distribution of the anion within the soil. Regarding homogenous NO_3_^−^ provision, total root length increases under moderate NO_3_^−^ supply, but decreases under extreme conditions, such as an excess or a severe deficiency. This reflects a systemic response that probably depends on plant nutritional status and hormonal balance [14,114]. Conversely, localized NO_3_^−^ availability directly stimulates the LR elongation and emergence into the NO_3_^−^-enriched-patch, while LR growth is inhibited outside that zone. These combined responses represent an adaptive strategy to cope with the spatial heterogeneity of NO_3_^−^ availability in soil (Figure 4) [32].

Needless to say, RSA changes require a wide reprogramming of gene expression in roots, which in Arabidopsis NRT1.1 plays a double role (Figure 1). At a null or low NO_3_^−^ concentration, NRT1.1 is phosphorylated and recruited into functional microdomains at the PM, where it facilitates auxin transport out of the LR primordia, hence preventing their outgrowth. At high NO_3_^−^ concentrations, non-phosphorylated NRT1.1 goes toward oligomerization that, inducing its endocytosis and secretion, triggers the suppression of auxin transport as well as the stimulation of signaling pathways for LR development, partly overlapped by the NPR [16,49,115,116]. In Arabidopsis, it was also proposed that NRT2.1 could participate in the suppression of LR initiation induced by high carbon (C)/N ratios (Figure 1) [114]. However, the network of systemic and local signals behind this response is not yet characterized and several aspects await elucidation [18].

Similarly, RSA is differently affected by a homogeneous supply or localized provisions of NH_4_^+^. When NH_4_^+^ is provided as a sole N form, especially under elevated supplies, typical responses consist in the alteration of root gravitropism and in the inhibition of elongation of PRs and LRs (Figure 4). The first is mainly associated with the disturbance of auxin distribution in the root apex, while the second one was related to apoplastic and intracellular pH changes, increase in reactive oxygen species (ROS) formation, and alterations in protein glycosylation [117]. The involvement of the auxin signal is also suggested by the observation that NRT1.1 takes part in NH_4_^+^ toxicity in the absence of NO_3_^−^ [118]. Conversely, localized NH_4_^+^ provision to N starved plants significantly promotes LR branching (Figure 4). It was recently demonstrated that this response is related to the interplay between apoplastic pH and auxin signal, especially as far as it regards higher-order LR branching. In detail, the influx of NH_4_^+^ facilitated by AMTs activates the PM H^+^-ATPase (Section 4.2) increasing the acidification of the root apoplast. This pH shift prompts protonated auxin to permeate across the PM into cortical and epidermal cells, finally stimulating the emergence of LR primordia [119]. Moreover, it was proposed that a signaling event mediated by AMT1;3 is required for these changes in RSA (Figure 1) [120]. Whether AMT1;3 acts as an actual transceptor or is a part of a more complex regulatory network is still to be elucidated [117]. Curiously, the observation that under excess NH_4_^+^ AMT1;3 amass into clusters internalized by clathrin-mediated endocytosis [121], resembles somehow the mechanism proposed for the NRT1.1 signaling functionality. Finally, it is possible to speculate that the specific and differential phosphorylation events through which AMT1;3 goes in response to NO_3_^−^ (Section 2.2) could be somehow involved in mediating the synergistic effects on RSA prompted by the co-provision of NO_3_^−^ and NH_4_^+^, consisting of induction of LR elongation for NO_3_^−^ and of LR formation for NH_4_^+^ (Figure 4).

Although root growth is inhibited by several amino acids, for most of them the effect was associated with indirect metabolic responses [12]. However, direct effects on RSA were proposed, among which that induced by Glu in Arabidopsis is the best characterized (Figure 4). Glu provision at low concentration (<50 µM) exerts an inhibitory effect on PR growth. It was shown that this response, especially in the early phase, is dependent on the perception of Glu by the PR tip, and is also combined with minor elongation of pre-formed LRs and with stimulation of outgrowth of new LRs behind the PR apex, finally resulting in a shorter and more branched root system [122]. This response involves auxin and kinase-mediated signals and was interpreted as an adaptive strategy to enhance plant competitiveness against microorganisms in amino acid uptake into enriched soil zones [122,123]. Interestingly, the effect of Glu on Arabidopsis root growth is strongly inhibited by NO_3_^−^, but not by other forms of N. The authors suggested that the NO_3_^−^ ion itself acts by an NRT1.1-mediated signal at the PR tip to repress the root architectural changes induced by Glu when inorganic N forms are abundant, conditions where a short and highly branched root system could be limiting [124]. A putative signaling role of amino acids in RSA and plant development was supported by the discovery of a family of Glutamate-like Receptors (GLRs) in plants. GLRs act as tetrameric amino-acid-gated Ca^2+^ channels involved in different aspects of plant physiology, including the regulation of C and N metabolism and abscisic acid signaling. However, their roles in RSA adaptation to amino acid provision still wait for elucidation [12]. Moreover, other amino acids seem to act in different ways, as for instance, the auxin effect attributed to tryptophan [122]. Finally, it is important to note that crop responses are species-specific and in soil the root system is exposed to a mix of different amino acids, which probably exert combined effects [10,125].

Overall, several proteomic studies provide evidence about the involvement of hormone response to different N nutrition. For instance, the analysis of the responses induced by NO_3_^−^ in maize roots suggested a down-regulation of the clathrin-mediated endocytosis that regulates the auxin signal and transport [126], as well as changes in abundance of enzymes involved in redox balance and nitric oxide (NO) signaling, were highlighted [126,127]. Moreover, a recent work-related the increase in elongation of roots occurring in rapeseed during N deficiency with a change in the abundance of an auxin-responsive protein and the reduction of peroxidase levels and activity. This RSA rearrangement was also associated with an enhanced abundance of several enzymes involved in cell wall organization and biogenesis, highlighting the importance of this metabolism in root growth [128]. Clearly, although these studies represent a good starting point, further work is needed to reach a complete description of this multifaceted process.

## 4. Other Activities in the Plant Cell Plasma Membrane Related to Nitrogen Uptake

### 4.1. Relations among Nitrogen Uptake and Aquaporins

In plants, aquaporins (AQPs) are protein channels, mediating the transport of water and a small set of solutes and gasses across cell membranes, with key roles in the physiological adaptations to abiotic and biotic conditions, including the availability of mineral nutrients [129]. AQPs belong to the superfamily of Major Intrinsic Proteins (MIPs) and in higher plants comprise five subfamilies, consisting of the Plasma membrane Intrinsic Proteins (PIPs), the Tonoplast Intrinsic Proteins (TIPs), the Nodulin26-like Intrinsic Proteins (NIP), the Small basic Intrinsic Proteins (SIPs), and the uncategorized (X) Intrinsic Proteins (XIPs) [130]. AQPs are small proteins containing six putative transmembrane domains and fold into the membrane with the C- and N-termini inside the cytosol. Specific motifs of the protein chain, some of which diverge among the five subfamilies, play a part in determining the substrate selectivity, as well as the internal loops being engaged in the AQP gating, a composite mechanism controlled by cytosolic pH, divalent cations, and phosphorylation. AQPs assemble into tetramers, in which each subunit forms a pore, plus a putative fifth pore formed in the center of the complex. Co-expression studies in *Xenopus* oocytes suggested the formation of heterotetramers whose composition could affect the targeting, pH sensitivity, and activity of PIPs [129]. For instance, in maize, it was demonstrated that interactions between members of the two subgroups PIP1 and PIP2 participate in targeting and stabilization of the water channel into the PM [131,132]. Interestingly, proteomic studies revealed different PTMs in PIP chains, such as multiple sites of phosphorylation detected in maize, *Brassica oleracea*, and Arabidopsis [133,134,135], as well as methylation events that in the last species were related to the trafficking of the protein [136].

The main AQPs in the root cell PM are PIPs, NIPs, and XIPs, among which PIPs show the highest selectivity and efficiency for water transport and play a predominant role in determining the root hydraulic conductivity (*L*p_r_) [129]. A significant increase in this parameter was observed in response to the renewed availability of NO_3_^−^, which probably is involved in sustaining the recovery of plant biomass accumulation and the nutrient uptake [129,137]. The studies aimed at associating this response to an up-regulation of the transcription of *PIP* genes in crops provided divergent results, probably due to different treatment timings (short- or long-period responses) and plant species [138,139]. Some explanations could be found in an excellent proteomic study that highlighted that during NO_3_^−^ deprivation the *L*p_r_ decrease scarcely correlates with the abundance of PIPs, especially for PIP1. On the contrary, the extent of phosphorylation seems to have a predominant role [140].

Since transpiration significantly enhances *L*p_r_ by increasing the *PIP* expression in roots [129], these responses may be a secondary effect induced by the recovery of leaf metabolism, as well as the osmotic effect of NO_3_^−^ cannot be excluded. However, a recent study conducted on Arabidopsis mutants showed that the *L*p_r_ correlates with the shoot NO_3_^−^ contents, and also revealed a side effect of the *NRT2.1* gene, uncoupled from its transport function, in the transcriptional and translational regulation of PIPs [141]. Considering the proposed signaling role for NRT2.1 during LR development [114], this finding opens the intriguing hypothesis of the interplay between NO_3_^−^ signaling, PIP expression, and RSA (Figure 1).

It was recently proposed that in plants AQPs could be also involved in determining the toxicity symptoms induced by excess NH_4_^+^ provision (Section 2.2) [57,59]. Starting from the hypothesis that in this condition an influx/efflux transport cycle of NH_3_/NH_4_^+^ across the PM of root cells occurs, it was recently demonstrated that the predominant form recruited in this futile cycle is the NH_3_ species. Moreover, the observed kinetic properties and responses to chemical treatments, such as the inhibitory effects of mercuric cation (Hg^2+^) and intracellular acidosis on NH_3_ cycling (known blockers of AQP activity [130]), were consistent with the implication of AQPs. According to the proposed model, AQPs passively mediate both NH_3_ influx and efflux across cell membranes, without energy dissipation, but allow NH_3_ to span into cell compartments on the basis of the concentration gradients and pH conditions, that result in the acidic trapping of NH_4_^+^ into the vacuole [59]. The hyper-accumulation of NH_4_^+^ into the vacuole could account for the decrease concentration of cations, especially K^+^, Mg^2+^, and Ca^2+^, which is considered one of the major causes for the “ammoniacal syndrome” in plants [9,142]. Interestingly, this model could in part explain why the co-provision of NO_3_^−^ and NH_4_^+^, balancing the cationic/anionic ratio into the vacuole, is the best nutritional condition [9]. Moreover, it is well known that NH_4_^+^ toxicity is rescued in plants under high K^+^ availability [142]. Although this is ascribable to a direct nutritional effect of K^+^ and competition against NH_4_^+^ transport (Section 2.2), since K^+^ availability is associated with a decrease in *L*p_r_ [129], it is intriguing to propose that a different AQP functionality could contribute to this effect (Figure 1). However, molecular aspects of the model require detailed elucidation. On the one hand it is now described that TIPs facilitate NH_3_ transport [143], on the other hand, to our knowledge, evidence of a significant contribution of PIPs and NIPs in the NH_3_ transport across the PM is lacking. Phosphoproteomics highlighted transient changes in the phosphorylation state of some PIP isoforms in Arabidopsis plants subjected to NH_4_^+^ resupply or deprivation [52,67]. Moreover, we detected that the abundance of two PIP2 isoforms in maize roots differently changed in response to NO_3_^−^ or NH_4_^+^ supply. Interestingly, in co-provision, NO_3_^−^ seemed to have a prevalent effect, opening new perspectives about the interplay between inorganic N forms and water homeostasis in roots [71].

Phylogenetic and structural analyses sorted the NIP subfamily into three main subgroups. Group I comprises members showing transport selectivity mainly for water and glycerol. Instead, NIP subgroup II shows high permeability to glycerol and large solutes, such as urea, but not to water, and, finally, group III is permeable to water and urea, but not glycerol [144,145]. Among the last two groups, NIP1 in zucchini (*Cucurbita pepo* L.) was the first one characterized as able to complement Dur3p deficiency (Section 2.4) in yeast [146]. In Arabidopsis roots, one of the most abundant members is NIP5;1, which is involved in boron (B) uptake, but, under B deficiency, it also participates in the high and low-affinity uptake of urea (Figure 1) [144,147]. This role for NIP was confirmed in different crops. For instance, in maize NIP2;1 and NIP2;4, both expressed in roots, were characterized as able to mediate urea transport [148]. A similar function was recently proposed for NIP2;1 in cucumber (*Cucumis sativus* L.), for which ectopic expression in *Arabidopsis thaliana* improved the growth of the wild-type genotype and rescued the growth of an *At*dur3 mutant, when urea was provided as the sole N source [145]. Overall, these results sustain the hypothesis that among the AQP family, some NIPs participate in urea uptake, even if other studies are needed to decipher the actual meanings for crop N nutrition and plant physiology.

### 4.2. The Involvement of Plasma Membrane H^+^-ATPase in Nitrogen Uptake

In plants, the plasma membrane H^+^-ATPase is a pump that exports cytosolic protons outside the PM, coupling the transport with ATP hydrolysis. Its activity generates the transmembrane electrochemical gradient that represents the driving force for solute import into plant cells, and hence it is intrinsically connected with nutrient uptake by roots. PM H^+^-ATPase is a large protein (of about 100 kDa) with 10 transmembrane domains and both N- and C-terminus protruding into the cytosolic face of the PM. The C-terminus acts as an auto-inhibitory regulatory domain, the phosphorylation of which at the penultimate Thr (Thr-955 in the model AHA2 of Arabidopsis) and the binding of 14-3-3 proteins lead to the activation of the enzyme [149]. In addition, multiple phosphorylation sites in the PM H^+^-ATPase were identified in planta, each with a different regulatory role as recently reviewed by Falhof and co-workers [150]. Considering that the uptake of NO_3_^−^, amino acids, and urea are all mediated by active solute/H^+^ symporters, the relevance of the PM H^+^-ATPase in the plant N nutrition is clear. Also, NH_4_^+^ uptake is associated with the stimulation of the PM H^+^-ATPase activity, triggered by the transient depolarization of PM electrical potential induced by the influx of the cation into the cytosol (Figure 1) [151]. Furthermore, the acidification of apoplast induced by NH_4_^+^ supply is involved in the typical responses in the RSA (Section 3).

Higher plants have multiple PM H^+^-ATPase isoforms, with tissue specificity and co-presence in a specific cell type. Phylogeny analysis of Arabidopsis PM H^+^-ATPase isoforms, named AHA (Autoinhibited H^+^-ATPase) recognizes five subfamilies. One group contains AHA4 and AHA11, the second one clusters isoforms similar to AHA1 (AHA2, AHA3, AHA5, AHA12), the third and the fourth only consist of AHA10 and AHA7, respectively, and the last group comprises AHA6, AHA8, and AHA9 [152,153]. Unexpectedly, AHA10-like isoforms target the tonoplast where they possibly assemble as hetero-oligomers [154,155]. Although this organization seems to be overall conserved across species, some plants were not recognized members for all the five subfamilies [27,153].

In Arabidopsis, AHA1, AHA2, and AHA7 are the predominant isoforms accumulated in root epidermal cells. Interestingly, AHA7 shows the peculiarity to be auto-inhibited by acidic extracellular pH, and, while AHA2 is fundamental in root cell expansion, AHA2 and AHA7 are required for limiting root hair length, a process in which they play different roles [153]. In the past years, phosphoproteomics highlighted multiple phosphorylated sites in the AHA1 and AHA2 isoforms in NH_4_^+^-adapted Arabidopsis plants. More recently, comparative proteomic analyses revealed a major accumulation of AHA2 under nitric than ammoniacal nutrition [156], overall confirming its involvement in the metabolic adaptations to the N external availability [52,67].

In this case, it is possible to draw reliable similitudes between Arabidopsis and some crops. For instance, a time-resolved analysis of the PM transport systems during PNR proposed that the PM H^+^-ATPase isoforms mainly involved are those encoded by *ZmHA2* and *ZmHA4* genes, both belonging to the second phylogenetic group [27]. Considering that a study of the root hair proteome in this crop revealed a reduction of AHA2 and AHA11 levels during N deprivation [157], it is possible to speculate that different isoforms play different roles in specific cell types.

**Figure 5:**
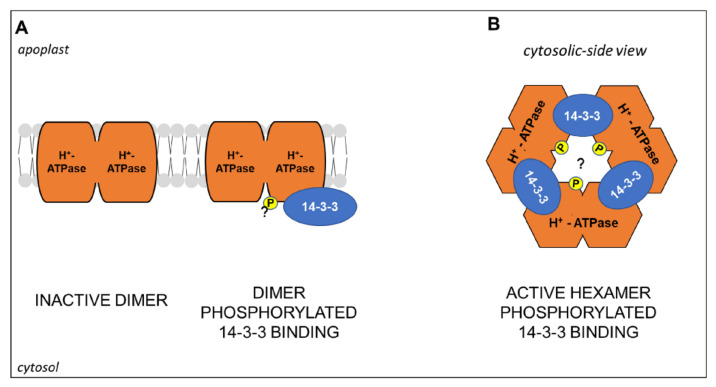
Schematic representation of (**A**) H^+^-ATPase/14-3-3 hexamer activation by an unknown kinase mediated-phosphorylation (“P”, yellow colored, question mark) and binding of 14-3-3 proteins. (**A**) Cytosolic-side view of the active hexameric complex. Model adapted from [159]. Further details are explained in the text.

To conclude, it is noteworthy that the PM H^+^-ATPase activity seems to be influenced not only at transcriptional levels and by phosphorylation but also by the oligomeric state of the enzyme. In tobacco cells, BN-PAGE (Blue Native, a non-denaturing PAGE) and electron microscopy analyses showed that in the microsomal fraction the enzyme exists in dimeric form, which assembles in hexameric complexes when phosphorylated and bound to 14-3-3 proteins [158]. Later, a model for the formation of the complex was proposed. Accordingly, a PM H^+^-ATPase dimer, inactive probably due to intermolecular contacts of the C-termini, after phosphorylation becomes a target for the binding of a 14-3-3 dimer, that allows sequential interactions with two other PM H^+^-ATPase/14-3-3 dimers, finally resulting in the assembly of active hexameric complexes (Figure 5) [159]. Interestingly, it was recently shown that in maize roots the PNR is associated with an increase in the abundance of monomeric, dimeric, and hexameric states of the PM H^+^-ATPase [27]. Overall, several questions are still open, such as those regarding the functional roles of different oligomeric states and the putative involvement of different isoforms, that could be effectively addressed in the future, also thanks to the recent technical improvements in plant proteomics (Section 5).

## 5. Nitrogen Nutrition in Plants and Root Proteomics: Goals and Pitfalls

In the last decades, proteomics was applied to study various aspects of N nutrition in plants, analyzing different kinds of samples, from whole seedlings to specific cell types, and combining different analytical strategies. We selected some studies devoted to investigating specifically the proteomic profiles of roots, conducted in Arabidopsis and crops (Table 2).

This selection includes studies that adopted different experimental plant growth conditions, such as availability and starvation of a specific N form, co-provision, re-supply, and depletion treatments. At a first look, it is evident that most of them deal with the plant responses to inorganic N forms, while those induced by organic N nutrients were much less frequently studied (Table 2). This trend mirrors the overall literature and indicates that the relations among plant nutrition and organic N forms represent a research field that could deserve attention in the next few years.

Despite individual topics, the studies in Table 2 are congruent in highlighting similar traits of root metabolism being affected by N availability. Some common traits include the reciprocal interplay between N and C metabolism, including protein synthesis and folding, in the relations between NH_4_^+^ assimilation and mitochondrial activities as well as the effects of N availability on cell redox homeostasis and peroxidase abundance in roots.

Among those reported in Table 2, each proteomic methodological approach has its specific advantages and pitfalls.

The first steps of sample preparation have a very high relevance in determining the features of the proteome subset under investigation. In the past years, most of the proteomic studies on N nutrition in plants analyzed total or soluble proteomes, especially in crops (Table 2). This strategy is particularly suitable to highlight the interplay among different metabolic pathways and allows to obtain a widespread holistic overview of the proteome. Hence, it is advantageous when the topic of interest is addressed by proteomics in a given plant species for the first time, as often occurs in crops. However, the analysis of entire proteomes at once greatly limits the detection of the less abundant proteins that frequently play key roles in the plant responses to nutritional stimuli, such as transcription factors, kinases and phosphatases, receptors and, also, transporters [164].

On the other hand, fractionation techniques allowing the purification of microsomal or plasma membrane enriched samples, significantly help in overcoming this drawback. To our knowledge, until now this strategy was adopted to study root responses to N nutrition only in Arabidopsis [52,67,140]. Coupled with phosphopeptide enrichment and gel-free protein quantification, it greatly contributed to the study of the roles of PTMs in the functionality of several transporters involved in N uptake, as extensively reported above.

Similarly, the selection of the best analytical techniques to resolve and quantify a plant proteomics sample mainly depends on the aims of the research. Gel-based proteomics is very suitable to individually analyze allelic variants and isoforms [165]. For instance, a 2-DE based approach pointed out different effects of NO_3_^−^ and/or NH_4_^+^ availability on the abundance and PTMs of the Glutamine Synthetase (GS) (iso)forms in maize roots [161]. However, conventional 2-DE protocols are not very suitable to resolve hydrophobic proteins and hence are poorly applicable in membrane proteomics [165]. Unfortunately, although a gel-free targeted proteomic methodology was defined to analyze the abundance of members of the NRT1, NRT2, AMT1, AHA, and PIP subfamilies in Arabidopsis [166], its application to other plant species still seems to be very laborious.

At the same time, the development of LC-MS/MS-based analytical techniques to quantify the proteins in complex samples, such as isobaric/isotopic labeling or the label-free shotgun approach, has greatly increased the number of proteins quantifiable in a single experiment. However, plant samples generally contain a high amount of compounds, such as pigments, lipids, polysaccharides, and secondary metabolites, which interfere with LC-MS/MS. Therefore, LC-MS/MS often gives the best results when combined with a prior purification of the proteome by SDS-PAGE [165]. This very powerful approach, called GeLC-MS, was recently applied to analyze the time course, within the first 54 h of induction, of the responses in roots and leaves of maize plants exposed to NO_3_^−^ and/or NH_4_^+^, allowing the simultaneous quantification of hundreds of proteins [71]. Another aspect that deserves attention is that overall shotgun approaches give information also about the components of the proteome not affected by the experimental treatments, that even if often overlooked, could be relevant from a biochemical point of view.

## 6. Conclusions and Future Trends

In the recent decades, proteomics gave its contribution in discerning the metabolic network and interactions on which N nutrition in plants relies on. However, it is evident that future work is needed to unravel this complexity in crops, and also in relation to organic N nutrition. Recently, deep proteomic profiling was applied with excellent results in several crops, including maize [167], wheat [168], and tomato [169], opening the way for their application in the study of plant responses to environmental stimuli, such as N nutrition. Similarly, considering the relevance of protein complexes in the functionality of the PM transporters (often highlighted in this review), the newly available approaches to analyze protein complexes [170] could be very effective in the future to achieve a better discerning of the role played by protein–protein interactions.

## Figures and Tables

**Table 1:** Transporters involved in N uptake in roots of *Arabidopsis thaliana*. The table reports the main information about transporters, family, affinity range, and tissue specificity in roots.

N Forms	Family	Protein	Affinity	Function	Tissue Specificity in Roots	Ref
**Nitrate**	**NPF**	**NRT1.1** (AtNPF6.3)	Dual	Uptake NO_3_^−^ transceptor Auxin transporter	Primary root tip, emerging lateral roots, epidermis near root tips, cortex, and endodermis in the mature portion of the root	[29,30,31,32]
		**NRT1.2** (AtNPF4.6)	Low	Uptake	Root hairs and epidermis	[33]
		**NAXT1** (AtNPF2.7)	Low	Efflux	Cortex of mature roots	[34]
		**NRT2.1**	High	Uptake NO_3_^−^ transceptor (?)	Epidermis, cortex, and endodermis in the mature portion of root	[32,35]
	**NRT2**	**NRT2.2**	High	Uptake	Root	[36]
		**NRT2.4**	High *	Uptake	Epidermis of lateral roots	[37]
		**NRT2.5**	High *	Uptake	Root hairs, epidermis, and cortex	[38]
**Ammonium**	**AMT1**	**AMT1;1**	High	Uptake	Root tip, root hairs, epidermis, and cortex	[39]
		**AMT1;2**	High	Retrieval from apoplast	Endodermis near the root hair differentiation zone, cortex in the zone of emerging lateral root	[40]
		**AMT1;3**	High	Uptake NH_4_^+^ transceptor (?)	Root tip, root hairs, epidermis, and cortex	[39]
		**AMT1;5**	High *	Uptake	Root tip, root hairs, epidermis	[40]
**Amino acids**	**LHT**	**LHT1**	High	Uptake of neutral and acidic amino acids, His	In young seedlings in the epidermis of later and emerging roots; in older plants, in root tip	[41,42]
		**LHT6**	High	Uptake of acidic amino acids, Gln, Ala, (Phe?)	Root hairs, epidermis, cortex, endodermis	[7]
	**AAP**	**AAP5**	High	Uptake of Arg and Lys	Cortex	[42,43]
		**AAP1**	High	Glu, Ala, Gln, Pro, Ser	Root tip, root hairs, epidermis, cortex, endodermis, and vascular cylinder	[7,44]
	**ProT**	**ProT2**	Low **	Pro and glycine betaine	Epidermis and cortex	[45]
**Urea**	**SSS**	**DUR3**	High	Uptake	Epidermis, cortex, vasculature tissues near the xylem	[46]

* very high affinity; ** the activity was tested only in high availability of Pro, to our knowledge, the high affinity could not be excluded. For acronyms see the text.

**Table 2:** Proteomic studies on plant responses to different N nutrient availabilities in roots. PepF: Peptide Fractionation. NO_3_^−^/NH_4_^+^ indicates co-provision. The other acronyms are detailed in the text. Plant species are reported in subtitles.

Proteome Fraction	Analytical Methods	N Nutrient	Major Results	Ref.
***Arabidopsis thaliana***				
Total	iTRAQ / PepF + LC-MS/MS	NO_3_^−^, NH_4_^+^	NO_3_^−^ or NH_4_^+^ availability differently affects C, N, and secondary metabolism, peroxidases, and AHA2.	[156]
Microsomal	PepF / TiO_2_ + LC-MS/MS	NO_3_^−^	NO_3_^−^ deprivation induces changes in abundance and PTMs of PIPs.	[140]
Microsomal	TiO_2_ + LC-MS/MS	NO_3_^−^, NH_4_^+^	N deprivation induces transient changes in the proteome and in phosphorylation of NRT2.1, AMT1;1, AMT1;3, DUR3, PIP2.2, PIP3, AHA1, AHA2.	[67]
PM	BN-PAGE + GeLC-MS/MS	NO_3_^−^, NO_3_^−^/NH_4_^+^	Regulative role of Ser-501 phosphorylation for the activity of NRT2.1.	[54]
***Brassica* spp.**				
Total	TMT / PepF + LC-MS/MS	N	N deficiency induces changes in the abundance of proteins involved in auxin and cell wall metabolism.	[128]
Total	2-DE + MALDI-MS/MS	NO_3_^−^, glycine	Glycine availability induces changes in the abundance of proteins involved in N metabolism and in defense.	[99]
***Zea mays***				
Total (root hair)	LC-MS/MS	NO_3_^−^/NH_4_^+^	N deprivation affects N, amino acid, and C metabolism and the abundance of peroxidases and AHA2 and AHA11.	[157]
Total	2-DE + MALDI-MS/MS	NO_3_^−^	NUE is associated with different responses of C and N metabolism to NO_3_^−^ availability.	[160]
Total (root transition zone)	iTRAQ + GeLC-MS/MS	NO_3_^−^	Relations of NO_3_^−^ supply with the abundance of peroxidases and of proteins involved in hormone balances.	[126]
Total	GeLC-MS/MS	NO_3_^−^, NH_4_^+^, NO_3_/NH_4_^+^	NO_3_^−^ and NH_4_^+^ availability differently affects N and C metabolism, protein synthesis, K^+^ channel, peroxidases, PIP2 isoforms.	[71]
Soluble	2-DE + LC-MS/MS	NO_3_^−^	NO_3_^−^ supply affects enzyme involved in N and C metabolism and in redox balance.	[127]
Soluble	2-DE + LC-MS/MS	NO_3_^−^, NH_4_^+^, NO_3_^−^/NH_4_^+^	NO_3_^−^ and NH_4_^+^ availability differently affects abundance and phosphorylation of GS (iso)forms.	[161]
***Hordeum vulgare***				
Soluble	2-DE + MALDI MS/MS	NO_3_^−^, NH_4_^+^	NO_3_^−^, NH_4_^+^ and N deficiency differently affects C, N metabolism, peroxidases, and redox balance.	[162]
***Lolium perenne***				
Soluble	2-DE + MALDI MS	NO_3_^−^ /NH_4_^+^, Gly	Glycine availability induces changes in abundances of enzymes involved in C, Met, and adenosine metabolism.	[98]
***Lycopersicon esculentum***				
Total	TMT/PepF + LC-MS/MS	NO_3_^−^, NH_4_^+^	NO_3_^−^ and NH_4_^+^ availability differently affects the abundance of enzymes involved in C and N metabolism and peroxidases.	[163]
***Solanum tuberosum***				
Total	LC-MS/MS	NO_3_^−^/NH_4_^+^	NUE is associated with different responses of C and N metabolism to NO_3_^−^ availability.	[72]

## References

[1] Hawkesford M., Horst W., Kichey T., Lambers H., Schjoerring J., Møller I.S., White P. (2012). Functions of Macronutrients. Marschner’s Mineral Nutrition of Higher Plants.

[2] Andrews M., Raven J.A., Lea P.J. (2013). Do Plants Need Nitrate? The Mechanisms by Which Nitrogen Form Affects Plants: Do Plants Need Nitrate?. Ann. Appl. Biol..

[3] Xu G., Fan X., Miller A.J. (2012). Plant Nitrogen Assimilation and Use Efficiency. Annu. Rev. Plant Biol..

[4] Bloom A. (2015). J The increasing importance of distinguishing among plant nitrogen sources. Curr. Opin. Plant Biol..

[5] Owen A.G., Jones D.L. (2001). Competition for Amino Acids between Wheat Roots and Rhizosphere Microorganisms and the Role of Amino Acids in Plant N Acquisition. Soil Biol. Biochem..

[6] Becker-Ritt A.B., Martinelli A.H.S., Mitidieri S., Feder V., Wassermann G.E., Santi L., Vainstein M.H., Oliveira J.T.A., Fiuza L.M., Pasquali G. (2007). Antifungal Activity of Plant and Bacterial Ureases. Toxicon.

[7] Perchlik M., Foster J., Tegeder M. (2014). Different and Overlapping Functions of Arabidopsis LHT6 and AAP1 Transporters in Root Amino Acid Uptake. J. Exp. Bot..

[8] Paungfoo-Lonhienne C., Visser J., Lonhienne T.G.A., Schmidt S. (2012). Past, Present and Future of Organic Nutrients. Plant Soil.

[9] Miller A.J., Cramer M.D. (2005). Root Nitrogen Acquisition and Assimilation. Plant Soil.

[10] Näsholm T., Kielland K., Ganeteg U. (2009). Uptake of Organic Nitrogen by Plants: Tansley Review. New Phytol..

[11] Yao X., Nie J., Bai R., Sui X. (2020). Amino Acid Transporters in Plants: Identification and Function. Plants.

[12] Forde B.G. (2014). Glutamate Signalling in Roots. J. Exp. Bot..

[13] Wang W.-H., Köhler B., Cao F.-Q., Liu L.-H. (2008). Molecular and Physiological Aspects of Urea Transport in Higher Plants. Plant Sci..

[14] Kiba T., Kudo T., Kojima M., Sakakibara H. (2011). Hormonal Control of Nitrogen Acquisition: Roles of Auxin, Abscisic Acid, and Cytokinin. J. Exp. Bot..

[15] Kraiser T., Gras D.E., Gutiérrez A.G., González B., Gutiérrez A.R. (2011). A holistic view of nitrogen acquisition in plants. J. Exp. Bot..

[16] Maghiaoui A., Gojon A., Bach L. (2020). NRT1.1-Centered Nitrate Signaling in Plants. J. Exp. Bot..

[17] Hao D.-L., Zhou J.-Y., Yang S.-Y., Qi W., Yang K.-J., Su Y.-H. (2020). Function and Regulation of Ammonium Transporters in Plants. Int. J. Mol. Sci..

[18] Gojon A., Krouk G., Perrine-Walker F., Laugier E. (2011). Nitrate Transceptor(s) in Plants. J. Exp. Bot..

[19] Miller A.J., Smith S.J. (1996). Nitrate Transport and Compartmentation in Cereal Root Cells. J. Exp. Bot..

[20] Crawford N.M., Glass A.D.M. (1998). Molecular and Physiological Aspects of Nitrate Uptake in Plants. Trends Plant Sci..

[21] Espen L. (2004). Effect of NO_3_^-^ Transport and Reduction on Intracellular pH: An in Vivo NMR Study in Maize Roots. J. Exp. Bot..

[22] Tsay Y.-F., Chiu C.-C., Tsai C.-B., Ho C.-H., Hsu P.-K. (2007). Nitrate Transporters and Peptide Transporters. FEBS Lett..

[23] Léran S., Varala K., Boyer J.-C., Chiurazzi M., Crawford N., Daniel-Vedele F., David L., Dickstein R., Fernandez E., Forde B. (2014). A Unified Nomenclature of NITRATE TRANSPORTER 1/PEPTIDE TRANSPORTER Family Members in Plants. Trends Plant Sci..

[24] Forde B.G. (2000). Nitrate Transporters in Plants: Structure, Function and Regulation. Biochim. Biophys. Acta Biomembr..

[25] Glass A.D.M. (2002). The Regulation of Nitrate and Ammonium Transport Systems in Plants. J. Exp. Bot..

[26] Scheible W.-R., Morcuende R., Czechowski T., Fritz C., Osuna D., Palacios-Rojas N., Schindelasch D., Thimm O., Udvardi M.K., Stitt M. (2004). Genome-Wide Reprogramming of Primary and Secondary Metabolism, Protein Synthesis, Cellular Growth Processes, and the Regulatory Infrastructure of Arabidopsis in Response to Nitrogen. Plant Physiol..

[27] Pii Y., Alessandrini M., Dall’Osto L., Guardini K., Prinsi B., Espen L., Zamboni A., Varanini Z. (2016). Time-Resolved Investigation of Molecular Components Involved in the Induction of NO_3_^−^ High Affinity Transport System in Maize Roots. Front. Plant Sci..

[28] Jacquot A., Li Z., Gojon A., Schulze W., Lejay L. (2017). Post-Translational Regulation of Nitrogen Transporters in Plants and Microorganisms. J. Exp. Bot..

[29] Ho C.-H., Lin S.-H., Hu H.-C., Tsay Y.-F. (2009). CHL1 Functions as a Nitrate Sensor in Plants. Cell.

[30] Liu K.-H., Tsay Y.-F. (2003). Switching between the Two Action Modes of the Dual-Affinity Nitrate Transporter CHL1 by Phosphorylation. EMBO J..

[31] Huang N.-C., Chiang C.-S., Crawford N.M., Tsay Y.-F. (1996). *CHL1* Encodes a Component of the Low-Affinity Nitrate Uptake System in Arabidopsis and Shows Cell Type-Specific Expression in Roots. Plant Cell.

[32] Remans T., Nacry P., Pervent M., Filleur S., Diatloff E., Mounier E., Tillard P., Forde B.G., Gojon A. (2006). The Arabidopsis NRT1.1 Transporter Participates in the Signaling Pathway Triggering Root Colonization of Nitrate-Rich Patches. Proc. Natl. Acad. Sci. USA.

[33] Huang N.-C., Liu K.-H., Lo H.-J., Tsay Y.-F. (1999). Cloning and Functional Characterization of an Arabidopsis Nitrate Transporter Gene That Encodes a Constitutive Component of Low-Affinity Uptake. Plant Cell.

[34] Segonzac C., Boyer J.-C., Ipotesi E., Szponarski W., Tillard P., Touraine B., Sommerer N., Rossignol M., Gibrat R. (2007). Nitrate Efflux at the Root Plasma Membrane: Identification of an *Arabidopsis* Excretion Transporter. Plant Cell.

[35] Chopin F., Wirth J., Dorbe M.-F., Lejay L., Krapp A., Gojon A., Daniel-Vedele F. (2007). The Arabidopsis Nitrate Transporter AtNRT2.1 Is Targeted to the Root Plasma Membrane. Plant Physiol. Biochem..

[36] Okamoto M., Vidmar J.J., Glass A.D.M. (2003). Regulation of NRT1 and NRT2 Gene Families of *Arabidopsis thaliana*: Responses to Nitrate Provision. Plant Cell Physiol..

[37] Kiba T., Feria-Bourrellier A.-B., Lafouge F., Lezhneva L., Boutet-Mercey S., Orsel M., Bréhaut V., Miller A., Daniel-Vedele F., Sakakibara H. (2012). The *Arabidopsis* Nitrate Transporter NRT2.4 Plays a Double Role in Roots and Shoots of Nitrogen-Starved Plants. Plant Cell.

[38] Lezhneva L., Kiba T., Feria-Bourrellier A.-B., Lafouge F., Zoufan P., Sakakibara H. (2014). The Arabidopsis Nitrate Transporter NRT2.5 Plays a Role in Nitrate Acquisition and Remobilization in Nitrogen-starved Plants. Plant J..

[39] Loqué D., Yuan L., Kojima S., Gojon A., Wirth J., Gazzarrini S., Ishiyama K., Takahashi H., von Wirén N. (2006). Additive Contribution of AMT1;1 and AMT1;3 to High-Affinity Ammonium Uptake across the Plasma Membrane of Nitrogen-Deficient Arabidopsis Roots. Plant J..

[40] Yuan L., Loqué D., Kojima S., Rauch S., Ishiyama K., Inoue E., Takahashi H., von Wirén N. (2007). The Organization of High-Affinity Ammonium Uptake in *Arabidopsis* Roots Depends on the Spatial Arrangement and Biochemical Properties of AMT1-Type Transporters. Plant Cell.

[41] Hirner A., Ladwig F., Stransky H., Okumoto S., Keinath M., Harms A., Frommer W.B., Koch W. (2006). *Arabidopsis* LHT1 Is a High-Affinity Transporter for Cellular Amino Acid Uptake in Both Root Epidermis and Leaf Mesophyll. Plant Cell.

[42] Svennerstam H., Jämtgård S., Ahmad I., Huss-Danell K., Näsholm T., Ganeteg U. (2011). Transporters in Arabidopsis Roots Mediating Uptake of Amino Acids at Naturally Occurring Concentrations. New Phytol..

[43] Svennerstam H., Ganeteg U., Näsholm T. (2008). Root Uptake of Cationic Amino Acids by Arabidopsis Depends on Functional Expression of Amino Acid Permease 5. New Phytol..

[44] Lee Y.-H., Foster J., Chen J., Voll L.M., Weber A.P.M., Tegeder M. (2007). AAP1 Transports Uncharged Amino Acids into Roots of Arabidopsis: Amino Acid Uptake by the Root. Plant J..

[45] Lehmann S., Gumy C., Blatter E., Boeffel S., Fricke W., Rentsch D. (2011). In Planta Function of Compatible Solute Transporters of the AtProT Family. J. Exp. Bot..

[46] Kojima S., Bohner A., Gassert B., Yuan L., Wirén N. (2007). von AtDUR3 Represents the Major Transporter for High-Affinity Urea Transport across the Plasma Membrane of Nitrogen-Deficient Arabidopsis Roots. Plant J..

[47] Sun J., Bankston J.R., Payandeh J., Hinds T.R., Zagotta W.N., Zheng N. (2014). Crystal Structure of the Plant Dual-Affinity Nitrate Transporter NRT1.1. Nature.

[48] Xu J., Li H.-D., Chen L.-Q., Wang Y., Liu L.-L., He L., Wu W.-H. (2006). A Protein Kinase, Interacting with Two Calcineurin B-like Proteins, Regulates K^+^ Transporter AKT1 in Arabidopsis. Cell.

[49] Bouguyon E., Brun F., Meynard D., Kubeš M., Pervent M., Leran S., Lacombe B., Krouk G., Guiderdoni E., Zažímalová E. (2015). Multiple Mechanisms of Nitrate Sensing by Arabidopsis Nitrate Transceptor NRT1.1. Nat. Plants.

[50] Yong Z., Kotur Z., Glass A.D.M. (2010). Characterization of an Intact Two-component High-affinity Nitrate Transporter from Arabidopsis Roots. Plant J..

[51] Kotur Z., Glass A.D.M. (2015). A 150 KDa Plasma Membrane Complex of AtNRT2.5 and AtNAR2.1 Is the Major Contributor to Constitutive High-Affinity Nitrate Influx in *Arabidopsis thaliana*. Plant Cell Environ..

[52] Engelsberger W.R., Schulze W.X. (2012). Nitrate and Ammonium Lead to Distinct Global Dynamic Phosphorylation Patterns When Resupplied to Nitrogen-starved Arabidopsis Seedlings. Plant J..

[53] Zou X., Liu M., Wu W., Wang Y. (2020). Phosphorylation at Ser28 Stabilizes the *Arabidopsis* Nitrate Transporter NRT2.1 in Response to Nitrate Limitation. J. Integr. Plant Biol..

[54] Jacquot A., Chaput V., Mauries A., Li Z., Tillard P., Fizames C., Bonillo P., Bellegarde F., Laugier E., Santoni V. (2020). NRT2.1 C-terminus Phosphorylation Prevents Root High Affinity Nitrate Uptake Activity in *Arabidopsis thaliana*. New Phytol..

[55] Wen Z., Tyerman S.D., Dechorgnat J., Ovchinnikova E., Dhugga K.S., Kaiser B.N. (2017). Maize NPF6 Proteins Are Homologs of Arabidopsis CHL1 That Are Selective for Both Nitrate and Chloride. Plant Cell.

[56] Feng H., Fan X., Yan M., Liu X., Miller A.J., Xu G. (2011). Multiple Roles of Nitrate Transport Accessory Protein NAR2 in Plants. Plant Signal. Behav..

[57] Esteban R., Ariz I., Cruz C., Moran J.F. (2016). Review: Mechanisms of Ammonium Toxicity and the Quest for Tolerance. Plant Sci..

[58] Loque D., von Wiren N. (2004). Regulatory Levels for the Transport of Ammonium in Plant Roots. J. Exp. Bot..

[59] Coskun D., Britto D.T., Li M., Becker A., Kronzucker H.J. (2013). Rapid Ammonia Gas Transport Accounts for Futile Transmembrane Cycling under NH_3_/NH_4_^+^ Toxicity in Plant Roots. Plant Physiol..

[60] Loqué D., Mora S.I., Andrade S.L.A., Pantoja O., Frommer W.B. (2009). Pore Mutations in Ammonium Transporter AMT1 with Increased Electrogenic Ammonium Transport Activity. J. Biol. Chem..

[61] Neuhäuser B., Ludewig U. (2014). Uncoupling of Ionic Currents from Substrate Transport in the Plant Ammonium Transporter *At* AMT1;2. J. Biol. Chem..

[62] Rawat S.R., Silim S.N., Kronzucker H.J., Siddiqi M.Y., Glass A.D.M. (1999). *AtAMT1* Gene Expression and NH_4_^+^ Uptake in Roots of *Arabidopsis thaliana*: Evidence for Regulation by Root Glutamine Levels. Plant J..

[63] Neuhäuser B., Dynowski M., Mayer M., Ludewig U. (2007). Regulation of NH_4_^+^ Transport by Essential Cross Talk between AMT Monomers through the Carboxyl Tails. Plant Physiol..

[64] Nühse T.S., Stensballe A., Jensen O.N., Peck S.C. (2004). Phosphoproteomics of the Arabidopsis Plasma Membrane and a New Phosphorylation Site Database. Plant Cell.

[65] Loqué D., Lalonde S., Looger L.L., von Wirén N., Frommer W.B. (2007). A Cytosolic Trans-Activation Domain Essential for Ammonium Uptake. Nature.

[66] Lanquar V., Loqué D., Hörmann F., Yuan L., Bohner A., Engelsberger W.R., Lalonde S., Schulze W.X., von Wirén N., Frommer W.B. (2009). Feedback Inhibition of Ammonium Uptake by a Phospho-Dependent Allosteric Mechanism in *Arabidopsis*. Plant Cell.

[67] Menz J., Li Z., Schulze W.X., Ludewig U. (2016). Early Nitrogen-deprivation Responses in Arabidopsis Roots Reveal Distinct Differences on Transcriptome and (Phospho-) Proteome Levels between Nitrate and Ammonium Nutrition. Plant J..

[68] Yuan L., Gu R., Xuan Y., Smith-Valle E., Loqué D., Frommer W.B., von Wirén N. (2013). Allosteric Regulation of Transport Activity by Heterotrimerization of *Arabidopsis* Ammonium Transporter Complexes in Vivo. Plant Cell.

[69] Straub T., Ludewig U., Neuhäuser B. (2017). The Kinase CIPK23 Inhibits Ammonium Transport in *Arabidopsis thaliana*. Plant Cell.

[70] Hoopen F.T., Cuin T.A., Pedas P., Hegelund J.N., Shabala S., Schjoerring J.K., Jahn T.P. (2010). Competition between Uptake of Ammonium and Potassium in Barley and Arabidopsis Roots: Molecular Mechanisms and Physiological Consequences. J. Exp. Bot..

[71] Prinsi B., Espen L. (2018). Time-Course of Metabolic and Proteomic Responses to Different Nitrate/Ammonium Availabilities in Roots and Leaves of Maize. Int. J. Mol. Sci..

[72] Jozefowicz A.M., Hartmann A., Matros A., Schum A., Mock H.-P. (2017). Nitrogen Deficiency Induced Alterations in the Root Proteome of a Pair of Potato (*Solanum Tuberosum* L.) Varieties Contrasting for Their Response to Low N. Proteomics.

[73] Wu X., Liu T., Zhang Y., Duan F., Neuhäuser B., Ludewig U., Schulze W.X., Yuan L. (2019). Ammonium and Nitrate Regulate NH_4_^+^ Uptake Activity of Arabidopsis Ammonium Transporter AtAMT1;3 via Phosphorylation at Multiple C-Terminal Sites. J. Exp. Bot..

[74] Gu R., Duan F., An X., Zhang F., von Wirén N., Yuan L. (2013). Characterization of AMT-Mediated High-Affinity Ammonium Uptake in Roots of Maize (*Zea mays* L.). Plant Cell Physiol..

[75] Noctor G., Novitskaya L., Lea P.J., Foyer C.H. (2002). Co-Ordination of Leaf Minor Amino Acid Contents in Crop Species: Significance and Interpretation. J. Exp. Bot..

[76] Okumoto S., Pilot G. (2011). Amino Acid Export in Plants: A Missing Link in Nitrogen Cycling. Mol. Plant.

[77] Pratelli R., Pilot G. (2014). Regulation of Amino Acid Metabolic Enzymes and Transporters in Plants. J. Exp. Bot..

[78] Jämtgård S., Näsholm T., Huss-Danell K. (2008). Characteristics of Amino Acid Uptake in Barley. Plant Soil.

[79] Biernath C., Fischer H., Kuzyakov Y. (2008). Root Uptake of N-Containing and N-Free Low Molecular Weight Organic Substances by Maize: A ^14^C/^15^N Tracer Study. Soil Biol. Biochem..

[80] Forsum O., Svennerstam H., Ganeteg U., Näsholm T. (2008). Capacities and Constraints of Amino Acid Utilization in Arabidopsis. New Phytol..

[81] Tegeder M., Rentsch D. (2010). Uptake and Partitioning of Amino Acids and Peptides. Mol. Plant.

[82] Chang A.B., Lin R., Studley W.K., Tran C.V., Saier M.H. (2004). Phylogeny as a Guide to Structure and Function of Membrane Transport Proteins. Mol. Membr. Biol..

[83] Jack D.L., Yang N.M.H., Saier M. (2001). The Drug/Metabolite Transporter Superfamily: The DMT Superfamily. Eur. J. Biochem..

[84] Ortiz-Lopez A., Chang H.-C., Bush D.R. (2000). Amino Acid Transporters in Plants. Biochim. Biophys. Acta.

[85] Phillips D.A., Fox T.C., King M.D., Bhuvaneswari T.V., Teuber L.R. (2004). Microbial Products Trigger Amino Acid Exudation from Plant Roots. Plant Physiol..

[86] Carvalhais L.C., Dennis P.G., Fedoseyenko D., Hajirezaei M.-R., Borriss R., von Wirén N. (2011). Root Exudation of Sugars, Amino Acids, and Organic Acids by Maize as Affected by Nitrogen, Phosphorus, Potassium, and Iron Deficiency. J. Plant Nutr. Soil Sci..

[87] Lesuffleur F., Paynel F., Bataillé M.-P., Le Deunff E., Cliquet J.-B. (2007). Root Amino Acid Exudation: Measurement of High Efflux Rates of Glycine and Serine from Six Different Plant Species. Plant Soil.

[88] Besnard J., Pratelli R., Zhao C., Sonawala U., Collakova E., Pilot G., Okumoto S. (2016). UMAMIT14 Is an Amino Acid Exporter Involved in Phloem Unloading in Arabidopsis Roots. J. Exp. Bot..

[89] Brady S.M., Orlando D.A., Lee J.-Y., Wang J.Y., Koch J., Dinneny J.R., Mace D., Ohler U., Benfey P.N. (2007). A High-Resolution Root Spatiotemporal Map Reveals Dominant Expression Patterns. Science.

[90] Fischer W.-N., Kwart M., Hummel S., Frommer W.B. (1995). Substrate Specificity and Expression Profile of Amino Acid Transporters (AAPs) in Arabidopsis. J. Biol. Chem..

[91] Tegeder M. (2012). Transporters for Amino Acids in Plant Cells: Some Functions and Many Unknowns. Curr. Opin. Plant Biol..

[92] Dinkeloo K., Boyd S., Pilot G. (2018). Update on Amino Acid Transporter Functions and on Possible Amino Acid Sensing Mechanisms in Plants. Semin. Cell Dev. Biol..

[93] Liu X., Bush D.R. (2006). Expression and Transcriptional Regulation of Amino Acid Transporters in Plants. Amino Acids.

[94] Lin Deng L.S. (2014). A Genome-Wide Analysis of the AAAP Gene Family in Maize. J. Proteom. Bioinform..

[95] Ma H., Cao X., Shi S., Li S., Gao J., Ma Y., Zhao Q., Chen Q. (2016). Genome-Wide Survey and Expression Analysis of the Amino Acid Transporter Superfamily in Potato (*Solanum tuberosum* L.). Plant Physiol. Biochem..

[96] Zhao Y., Xu Y., Wang Z., Zhang J., Chen X., Li Z., Li Z., Jin L., Wei P., Zhang L. (2017). Genome-Wide Identification and Characterization of an Amino Acid Permease Gene Family in *Nicotiana tabacum*. RSC Adv..

[97] Wan Y., King R., Mitchell R.A.C., Hassani-Pak K., Hawkesford M.J. (2017). Spatiotemporal Expression Patterns of Wheat Amino Acid Transporters Reveal Their Putative Roles in Nitrogen Transport and Responses to Abiotic Stress. Sci. Rep..

[98] Thornton B., Osborne S.M., Paterson E., Cash P. (2007). A Proteomic and Targeted Metabolomic Approach to Investigate Change in *Lolium perenne* Roots When Challenged with Glycine. J. Exp. Bot..

[99] Wang X., Tang D., Huang D. (2014). Proteomic Analysis of Pakchoi Leaves and Roots under Glycine–Nitrogen Conditions. Plant Physiol. Biochem..

[100] Liao Q., Tang T., Zhou T., Song H., Hua Y., Zhang Z. (2020). Integrated Transcriptional and Proteomic Profiling Reveals Potential Amino Acid Transporters Targeted by Nitrogen Limitation Adaptation. Int. J. Mol. Sci..

[101] Glibert P.M., Harrison J., Heil C., Seitzinger S. (2006). Escalating Worldwide Use of Urea—A Global Change Contributing to Coastal Eutrophication. Biogeochemistry.

[102] Kojima S., Bohner A., von Wirén N. (2006). Molecular Mechanisms of Urea Transport in Plants. J. Membr. Biol.

[103] Witte C.-P. (2011). Urea Metabolism in Plants. Plant Sci..

[104] Liu L.-H., Ludewig U., Frommer W.B., von Wirén N. (2003). AtDUR3 Encodes a New Type of High-Affinity Urea/H ^+^ Symporter in Arabidopsis. Plant Cell.

[105] Mérigout P., Lelandais M., Bitton F., Renou J.-P., Briand X., Meyer C., Daniel-Vedele F. (2008). Physiological and Transcriptomic Aspects of Urea Uptake and Assimilation in Arabidopsis Plants. Plant Physiol..

[106] Pinton R., Tomasi N., Zanin L. (2016). Molecular and Physiological Interactions of Urea and Nitrate Uptake in Plants. Plant Signal. Behav..

[107] Zanin L., Tomasi N., Wirdnam C., Meier S., Komarova N.Y., Mimmo T., Cesco S., Rentsch D., Pinton R. (2014). Isolation and Functional Characterization of a High Affinity Urea Transporter from Roots of *Zea mays*. BMC Plant Biol..

[108] Liu G.-W., Sun A.-L., Li D.-Q., Athman A., Gilliham M., Liu L.-H. (2015). Molecular Identification and Functional Analysis of a Maize (*Zea mays*) DUR3 Homolog That Transports Urea with High Affinity. Planta.

[109] Lupini A., Princi M.P., Araniti F., Miller A.J., Sunseri F., Abenavoli M.R. (2017). Physiological and Molecular Responses in Tomato under Different Forms of N Nutrition. J. Plant Physiol..

[110] Zanin L., Tomasi N., Zamboni A., Varanini Z., Pinton R. (2015). The Urease Inhibitor NBPT Negatively Affects DUR3-Mediated Uptake and Assimilation of Urea in Maize Roots. Front. Plant Sci..

[111] Lynch J. (1995). Root Architecture and Plant Productivity. Plant Physiol..

[112] Osmont K.S., Sibout R., Hardtke C.S. (2007). Hidden Branches: Developments in Root System Architecture. Annu. Rev. Plant Biol..

[113] Giehl R.F.H., Gruber B.D., von Wirén N. (2014). It’s Time to Make Changes: Modulation of Root System Architecture by Nutrient Signals. J. Exp. Bot..

[114] Zhang H., Rong H., Pilbeam D. (2007). Signalling Mechanisms Underlying the Morphological Responses of the Root System to Nitrogen in *Arabidopsis thaliana*. J. Exp. Bot..

[115] Krouk G., Lacombe B., Bielach A., Perrine-Walker F., Malinska K., Mounier E., Hoyerova K., Tillard P., Leon S., Ljung K. (2010). Nitrate-Regulated Auxin Transport by NRT1.1 Defines a Mechanism for Nutrient Sensing in Plants. Dev. Cell.

[116] Zhang X., Cui Y., Yu M., Su B., Gong W., Baluška F., Komis G., Šamaj J., Shan X., Lin J. (2019). Phosphorylation-Mediated Dynamics of Nitrate Transceptor NRT1.1 Regulate Auxin Flux and Nitrate Signaling in Lateral Root Growth. Plant Physiol..

[117] Liu Y., von Wirén N. (2017). Ammonium as a Signal for Physiological and Morphological Responses in Plants. J. Exp. Bot..

[118] Hachiya T., Noguchi K. (2011). Mutation of NRT1.1 Enhances Ammonium/Low pH-Tolerance in *Arabiopsis thaliana*. Plant Signal. Behav..

[119] Meier M., Liu Y., Lay-Pruitt K.S., Takahashi H., von Wirén N. (2020). Auxin-Mediated Root Branching Is Determined by the Form of Available Nitrogen. Nat. Plants.

[120] Lima J.E., Kojima S., Takahashi H., von Wirén N. (2010). Ammonium Triggers Lateral Root Branching in *Arabidopsis* in an AMMONIUM TRANSPORTER1;3-Dependent Manner. Plant Cell.

[121] Wang Q., Zhao Y., Luo W., Li R., He Q., Fang X., Michele R.D., Ast C., von Wiren N., Lin J. (2013). Single-Particle Analysis Reveals Shutoff Control of the Arabidopsis Ammonium Transporter AMT1;3 by Clustering and Internalization. Proc. Natl. Acad. Sci. USA.

[122] Walch-Liu P., Liu L.-H., Remans T., Tester M., Forde B.G. (2006). Evidence That L-Glutamate Can Act as an Exogenous Signal to Modulate Root Growth and Branching in *Arabidopsis thaliana*. Plant Cell Physiol..

[123] Forde B.G., Cutler S.R., Zaman N., Krysan P.J. (2013). Glutamate Signalling via a MEKK1 Kinase-Dependent Pathway Induces Changes in Arabidopsis Root Architecture. Plant J..

[124] Walch-Liu P., Forde B.G. (2008). Nitrate Signalling Mediated by the NRT1.1 Nitrate Transporter Antagonises L-Glutamate-Induced Changes in Root Architecture. Plant J..

[125] Canarini A., Kaiser C., Merchant A., Richter A., Wanek W. (2019). Root Exudation of Primary Metabolites: Mechanisms and Their Roles in Plant Responses to Environmental Stimuli. Front. Plant Sci..

[126] Trevisan S., Manoli A., Ravazzolo L., Botton A., Pivato M., Masi A., Quaggiotti S. (2015). Nitrate Sensing by the Maize Root Apex Transition Zone: A Merged Transcriptomic and Proteomic Survey. J. Exp. Bot..

[127] Prinsi B., Negri A.S., Pesaresi P., Cocucci M., Espen L. (2009). Evaluation of Protein Pattern Changes in Roots and Leaves of *Zea mays* Plants in Response to Nitrate Availability by Two-Dimensional Gel Electrophoresis Analysis. BMC Plant Biol..

[128] Qin L., Walk T.C., Han P., Chen L., Zhang S., Li Y., Hu X., Xie L., Yang Y., Liu J. (2019). Adaption of Roots to Nitrogen Deficiency Revealed by 3D Quantification and Proteomic Analysis. Plant Physiol..

[129] Maurel C., Boursiac Y., Luu D.-T., Santoni V., Shahzad Z., Verdoucq L. (2015). Aquaporins in Plants. Physiol. Rev..

[130] Singh R.K., Deshmukh R., Muthamilarasan M., Rani R., Prasad M. (2020). Versatile Roles of Aquaporin in Physiological Processes and Stress Tolerance in Plants. Plant Physiol. Biochem..

[131] Fetter K., Van Wilder V., Moshelion M., Chaumont F. (2004). Interactions between Plasma Membrane Aquaporins Modulate Their Water Channel Activity. Plant Cell.

[132] Zelazny E., Borst J.W., Muylaert M., Batoko H., Hemminga M.A., Chaumont F. (2007). FRET Imaging in Living Maize Cells Reveals That Plasma Membrane Aquaporins Interact to Regulate Their Subcellular Localization. Proc. Natl. Acad. Sci. USA.

[133] Van Wilder V., Miecielica U., Degand H., Derua R., Waelkens E., Chaumont F. (2008). Maize Plasma Membrane Aquaporins Belonging to the PIP1 and PIP2 Subgroups Are in Vivo Phosphorylated. Plant Cell Physiol..

[134] Casado-Vela J., Muries B., Carvajal M., Iloro I., Elortza F., Martínez-Ballesta M.C. (2010). Analysis of Root Plasma Membrane Aquaporins from *Brassica oleracea*: Post-Translational Modifications, de Novo Sequencing and Detection of Isoforms by High Resolution Mass Spectrometry. J. Proteome Res..

[135] Santoni V., Vinh J., Pflieger D., Sommerer N., Maurel C. (2003). A Proteomic Study Reveals Novel Insights into the Diversity of Aquaporin Forms Expressed in the Plasma Membrane of Plant Roots. Biochem. J..

[136] Santoni V., Verdoucq L., Sommerer N., Vinh J., Pflieger D., Maurel C. (2006). Methylation of Aquaporins in Plant Plasma Membrane. Biochem. J..

[137] Gorska A., Ye Q., Holbrook N.M., Zwieniecki M.A. (2008). Nitrate Control of Root Hydraulic Properties in Plants: Translating Local Information to Whole Plant Response. Plant Physiol..

[138] Gorska A., Zwieniecka A., Michele Holbrook N., Zwieniecki M.A. (2008). Nitrate Induction of Root Hydraulic Conductivity in Maize Is Not Correlated with Aquaporin Expression. Planta.

[139] Wang Y.-H., Garvin D.F., Kochian L.V. (2001). Nitrate-Induced Genes in Tomato Roots. Array Analysis Reveals Novel Genes That May Play a Role in Nitrogen Nutrition. Plant Physiol..

[140] di Pietro M., Vialaret J., Li G.-W., Hem S., Prado K., Rossignol M., Maurel C., Santoni V. (2013). Coordinated Post-Translational Responses of Aquaporins to Abiotic and Nutritional Stimuli in Arabidopsis Roots. Mol. Cell. Proteom..

[141] Li G., Tillard P., Gojon A., Maurel C. (2016). Dual Regulation of Root Hydraulic Conductivity and Plasma Membrane Aquaporins by Plant Nitrate Accumulation and High-Affinity Nitrate Transporter NRT2.1. Plant Cell Physiol..

[142] Britto D.T., Kronzucker H.J. (2002). NH_4_^+^ Toxicity in Higher Plants: A Critical Review. J. Plant Physiol..

[143] Loqué D., Ludewig U., Yuan L., von Wirén N. (2005). Tonoplast Intrinsic Proteins AtTIP2;1 and AtTIP2;3 Facilitate NH_3_ Transport into the Vacuole. Plant Physiol..

[144] Wallace I.S., Choi W.-G., Roberts D.M. (2006). The Structure, Function and Regulation of the Nodulin 26-like Intrinsic Protein Family of Plant Aquaglyceroporins. Biochim. Biophys. Acta Biomembr..

[145] Zhang L., Yan J., Vatamaniuk O.K., Du X. (2016). CsNIP2;1 Is a Plasma Membrane Transporter from *Cucumis sativus* That Facilitates Urea Uptake When Expressed in *Saccharomyces cerevisiae* and *Arabidopsis thaliana*. Plant Cell Physiol..

[146] Klebl F., Wolf M., Sauer N. (2003). A Defect in the Yeast Plasma Membrane Urea Transporter Dur3p Is Complemented by *CpNIP1*, a Nod26-like Protein from Zucchini (*Cucurbita pepo* L.), and by *Arabidopsis thaliana* δ-TIP or γ-TIP. FEBS Lett..

[147] Yang H., Menz J., Häussermann I., Benz M., Fujiwara T., Ludewig U. (2015). High and Low Affinity Urea Root Uptake: Involvement of NIP5;1. Plant Cell Physiol..

[148] Gu R., Chen X., Zhou Y., Yuan L. (2012). Isolation and Characterization of Three Maize Aquaporin Genes, *ZmNIP2;1*, *ZmNIP2;4* and *ZmTIP4;4* Involved in Urea Transport. BMB Rep..

[149] Palmgren M.G. (2001). Plant Plasma Membrane H^+^-ATPases: Powerhouses for Nutrient Uptake. Annu. Rev. Plant. Physiol. Plant. Mol. Biol..

[150] Falhof J., Pedersen J.T., Fuglsang A.T., Palmgren M. (2016). Plasma Membrane H^+^-ATPase Regulation in the Center of Plant Physiology. Mol. Plant.

[151] Schubert S., Yan F. (1997). Nitrate and Ammonium Nutrition of Plants: Effects on Acid/Base Balance and Adaptation of Root Cell Plasmalemma H^+^-ATPase. Z. Pflanz. Bodenk.

[152] Arango M., Gévaudant F., Oufattole M., Boutry M. (2003). The Plasma Membrane Proton Pump ATPase: The Significance of Gene Subfamilies. Planta.

[153] Hoffmann R.D., Olsen L.I., Ezike C.V., Pedersen J.T., Manstretta R., López-Marqués R.L., Palmgren M. (2019). Roles of Plasma Membrane Proton ATPases AHA2 and AHA7 in Normal Growth of Roots and Root Hairs in *Arabidopsis thaliana*. Physiol. Plant..

[154] Aprile A., Federici C., Close T.J., De Bellis L., Cattivelli L., Roose M.L. (2011). Expression of the H^+^-ATPase AHA10 Proton Pump Is Associated with Citric Acid Accumulation in Lemon Juice Sac Cells. Funct. Integr. Genom..

[155] Faraco M., Spelt C., Bliek M., Verweij W., Hoshino A., Espen L., Prinsi B., Jaarsma R., de Boher A.H., Di Sansebastiano G.-P. (2014). Hyperacidification of Vacuoles by the Combined Action of Two Different P-ATPases in the Tonoplast Determines Flower Color. Cell Rep..

[156] Coleto I., Vega-Mas I., Glauser G., González-Moro M., Marino D., Ariz I. (2019). New Insights on *Arabidopsis thaliana* Root Adaption to Ammonium Nutrition by the Use of a Quantitative Proteomic Approach. Int. J. Mol. Sci..

[157] Li Z., Phillip D., Neuhäuser B., Schulze W.X., Ludewig U. (2015). Protein Dynamics in Young Maize Root Hairs in Response to Macro- and Micronutrient Deprivation. J. Proteome Res..

[158] Kanczewska J., Marco S., Vandermeeren C., Maudoux O., Rigaud J.-L., Boutry M. (2005). Activation of the Plant Plasma Membrane H^+^-ATPase by Phosphorylation and Binding of 14-3-3 Proteins Converts a Dimer into a Hexamer. Proc. Natl. Acad. Sci. USA.

[159] Ottmann C., Marco S., Jaspert N., Marcon C., Schauer N., Weyand M., Vandermeeren C., Duby G., Boutry M., Wittinghofer A. (2007). Structure of a 14-3-3 Coordinated Hexamer of the Plant Plasma Membrane H^+^-ATPase by Combining X-Ray Crystallography and Electron Cryomicroscopy. Mol. Cell.

[160] Jin X., Li W., Hu D., Shi X., Zhang X., Zhang F., Fu Z., Ding D., Liu Z., Tang J. (2015). Biological Responses and Proteomic Changes in Maize Seedlings under Nitrogen Deficiency. Plant Mol. Biol. Rep..

[161] Prinsi B., Espen L. (2015). Mineral Nitrogen Sources Differently Affect Root Glutamine Synthetase Isoforms and Amino Acid Balance among Organs in Maize. BMC Plant Biol..

[162] Møller A.L.B., Pedas P., Andersen B., Svensson B., Schjoerring J.K., Finnie C. (2011). Responses of Barley Root and Shoot Proteomes to Long-Term Nitrogen Deficiency, Short-Term Nitrogen Starvation and Ammonium: N Responses of Barley Shoot and Root Proteomes. Plant Cell Environ..

[163] Xun Z., Guo X., Li Y., Wen X., Wang C., Wang Y. (2020). Quantitative Proteomics Analysis of Tomato Growth Inhibition by Ammonium Nitrogen. Plant Physiol. Biochem..

[164] Millar A.H., Taylor N.L. (2014). Subcellular Proteomics—Where Cell Biology Meets Protein Chemistry. Front. Plant Sci..

[165] Jorrin-Novo J.V., Komatsu S., Sanchez-Lucas R., Rodríguez de Francisco L.E. (2019). Gel Electrophoresis-Based Plant Proteomics: Past, Present, and Future. Happy 10th Anniversary Journal of Proteomics!. J. Proteom..

[166] Monneuse J.-M., Sugano M., Becue T., Santoni V., Hem S., Rossignol M. (2011). Towards the Profiling of the *Arabidopsis thaliana* Plasma Membrane Transportome by Targeted Proteomics. Proteomics.

[167] Walley J.W., Sartor R.C., Shen Z., Schmitz R.J., Wu K.J., Urich M.A., Nery J.R., Smith L.G., Schnable J.C., Ecker J.R. (2016). Integration of Omic Networks in a Developmental Atlas of Maize. Science.

[168] Duncan O., Trösch J., Fenske R., Taylor N.L., Millar A.H. (2017). Resource: Mapping the *Triticum aestivum* Proteome. Plant J..

[169] Szymanski J., Levin Y., Savidor A., Breitel D., Chappell-Maor L., Heinig U., Töpfer N., Aharoni A. (2017). Label-Free Deep Shotgun Proteomics Reveals Protein Dynamics during Tomato Fruit Tissues Development. Plant J..

[170] Aryal U.K., Xiong Y., McBride Z., Kihara D., Xie J., Hall M.C., Szymanski D.B. (2014). A Proteomic Strategy for Global Analysis of Plant Protein Complexes. Plant Cell.

